# Microbial Gatekeepers of Fertility in the Female Reproductive Microbiome of Cattle

**DOI:** 10.3390/ijms252010923

**Published:** 2024-10-10

**Authors:** Mounir Adnane, Aspinas Chapwanya

**Affiliations:** 1 Department of Biomedicine, Institute of Veterinary Sciences, University of Tiaret, Tiaret 14000, Algeria; mounir.adnane@univ-tiaret.dz; 2 Department of Clinical Sciences, Ross University School of Veterinary Medicine, Basseterre 00265, Saint Kitts and Nevis

**Keywords:** genital microbiome, fertility, metagenomics, dysbiosis, diseases, cattle

## Abstract

This review paper delves into the intricate relationship between the genital microbiome and fertility outcomes in livestock, with a specific focus on cattle. Drawing upon insights derived from culture-independent metagenomics studies, the paper meticulously examines the composition and dynamics of the genital microbiome. Through advanced techniques such as high-throughput sequencing, the review illuminates the temporal shifts in microbial communities and their profound implications for reproductive health. The analysis underscores the association between dysbiosis—an imbalance in microbial communities—and the development of reproductive diseases, shedding light on the pivotal role of microbial gatekeepers in livestock fertility. Furthermore, the paper emphasizes the need for continued exploration of uncharted dimensions of the female reproductive microbiome to unlock new insights into its impact on fertility. By elucidating the complex interplay between microbial communities and reproductive health, this review underscores the importance of innovative strategies aimed at enhancing fertility and mitigating reproductive diseases in livestock populations.

## 1. Introduction

The bacterial constituents within the genital tract during pivotal reproductive milestones such as pregnancy, metritis, and estrous cycles profoundly influence the animal’s wellbeing and fertility.

The bovine vagina harbors a complex and dynamic microbial community consisting of aerobic, facultative anaerobic, and anaerobic bacteria. This intricate blend functions as a natural defense mechanism, preventing the unchecked growth of pathogenic microorganisms [1]. What is intriguing is the variability observed in the dominant colonizing species across different studies, highlighting the dynamic nature of this ecosystem. Amidst this constant flux, new strains are continuously introduced, contributing to microbial diversity. Among the common inhabitants of the bovine vaginal tract are *Streptococcus* sp., *Staphylococcus* sp., *Enterococci*, and members of Enterobacteriaceae [2]. However, the dominance within this microbial landscape shifts, with species like *Aggregatibacter* sp., *Streptobacillus* sp., *Phocoenobacter* sp., *Sediminicola* sp., and *Sporobacter* sp. standing out as prevalent constituents in the vaginal microbiota of cows [3]. This dynamic interplay underscores the complexity of microbial colonization within the bovine reproductive tract. The most abundant phyla in the vaginal microbiota of dairy cattle are Firmicutes, Proteobacteria, Bacteroidetes, and Actinobacteria [4,5,6]. Some studies have also reported the presence of Tenericutes and Fusobacteria as significant components [7,8,9,10]. Additionally, the dominant fungal genus identified is *Mycosphaerella*, highlighting the diverse microbial landscape within the dairy cattle reproductive tract [6]. Recent studies have identified pathogenic fungi, particularly yeast and Penicillium species, as notable colonizers of the cervicovaginal fluids in Holstein dairy cattle [11]. These fungal pathogens have been increasingly recognized for their potential impact on reproductive health, adding complexity to the microbial ecosystem within the reproductive tract [12].

The cervix serves as a key anatomical barrier in the reproductive tract, and its associated microbiome plays an essential role in maintaining uterine health [13]. Recent advances in microbiome research, particularly through next-generation sequencing (NGS), have uncovered a far more complex and diverse bacterial population within the cervix than previously recognized using traditional culture-based methods [14]. The cervical microbiome, which includes dominant phyla such as Proteobacteria, Bacteroidetes, and Firmicutes, mirrors the diversity seen in the vaginal microbiome and fluctuates across different reproductive stages, including the clinical formative, gestation, and postpartum periods [15]. Studies have shown that disruptions in the cervical microbiome, particularly the overrepresentation of certain pathogenic bacteria like *Staphylococcus aureus* and higher levels of Bacteroidetes and Fusobacteria, are associated with reproductive diseases like metritis [16]. These microbial shifts are indicative of the cervix’s dynamic environment, which can be altered by infection, inflammation, or postpartum complications [17]. Understanding these changes in microbial composition is crucial for identifying potential biomarkers of reproductive disorders and developing targeted interventions to restore microbial balance [18].

The uterine microbiome, while primarily originating from the vagina, also includes microbes from the skin and gut [13,19]. However, the uterine microbiome is not as diverse as the vaginal microbiome. In a healthy uterus, Bacteroidetes, Fusobacteria, and Actinobacteria are often detected [20]. The genital microbiome’s composition undergoes marked fluctuations throughout the bovine life cycle, influenced by factors such as anatomical part, age, cyclicity, vaginal pH dynamics, nutrition, and farming conditions. Hormonal profile oscillations, particularly in estrogen and progesterone levels, significantly affect the abundance and diversity of the bacterial community during distinct phases of the estrous cycle [21]. Heightened Firmicutes levels in the vaginal milieu are primarily associated with diminished progesterone concentrations, while increased Proteobacteria abundance correlates with elevated progesterone levels [22]. These observations highlight the nuanced interplay between circulating steroid hormones and the dynamics of the vaginal microbiota in bovine reproductive physiology.

The aim of this review paper is to meticulously dissect and elucidate the intricate interplay between the female reproductive microbiome and fertility outcomes in cattle. By synthesizing data from diverse studies, the paper seeks to unravel the complex composition and dynamic shifts of microbial communities within the genital tract, exploring their profound impact on reproductive health. The objective is to provide a comprehensive analysis of how microbial diversity and dysbiosis influence key reproductive metrics, such as conception rates, pregnancy success, and the prevalence of reproductive disorders. Additionally, the paper aims to critically evaluate and contrast the methodologies employed in microbiome research, highlighting how different techniques shape the understanding of microbial influences. In compiling the references for this review, a rigorous approach was adopted to ensure the inclusion of relevant and high-quality studies. The literature search was conducted using major academic databases, including Springer, Elsevier, and Wiley, and scientific search engines such as Pubmed and Google Scholar with a focus on keywords such as “cow microbiome”, “genital microbiome”, “vagina”, “uterus”, “genital tract”, “bacteria”, “fertility”, and “reproduction”. These keywords were utilized in various combinations to capture a broad spectrum of pertinent research. The selection process emphasized relevance to the topic, recent publication dates, and clarity in methodology or synthesis. While this review is not systematic, the references were carefully chosen to provide a balanced and comprehensive overview, minimizing bias and ensuring that the insights presented are grounded in well-conducted and applicable research.

## 2. Microbial Composition in the Female Reproductive Tract

### 2.1. Taxonomic Diversity

**Figure 1:**
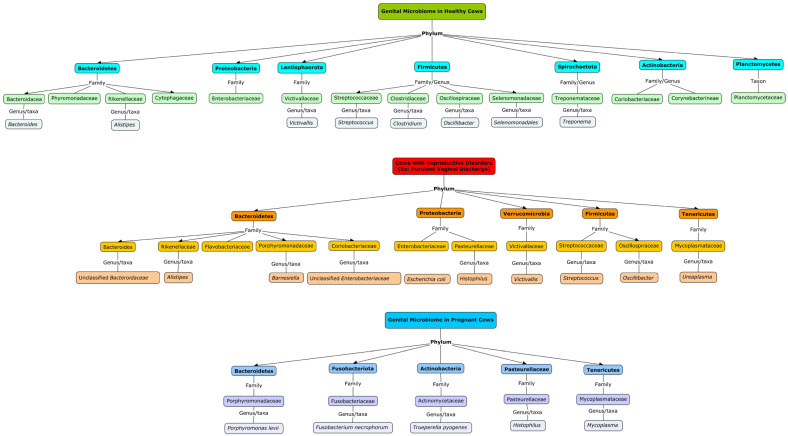
Conceptual map of vaginal and uterine microbiome composition in cows. It provides a visual representation of the microbiome composition in cows, focusing on differences based on health status and gestational stage. It categorizes bacteria into phyla, families, and genera, illustrating dominant and notable microbial communities found in cows without uterine infections, cows with reproductive disorders, and cows during gestation. It integrates findings from various studies to compare microbial dynamics, offering insights into the associations between microbiome diversity, dysbiosis, and reproductive health in cattle.

In cows without uterine infections, the vaginal microbiome is characterized by 15 taxa, with Bacteroidaceae (28.3%) and Enterobacteriaceae (17.8%) predominating, alongside notable presence of Victivallaceae (7.2%), Streptococcaceae (6.1%), Selenomonadaceae and Spirochaetaceae (5.6%), Porphyromonadaceae (5.6%), Rikenellaceae (3.9%), Coriobacteriaceae (3.3%), Clostridiaceae (3.3%), and Betaproteobacteria, Corynebacterineae, Cytophagaceae, Ruminococcaceae, and Planctomycetaceae (2.8%, each) [23] (Figure 1).

Conversely, cows afflicted with reproductive disorders such as purulent vaginal discharge exhibit a more diverse vaginal microbiome, comprising 68 taxa, with Bacteroidaceae (35.83%) and Enterobacteriaceae (18.62%) dominating, along with Pasteurellaceae, Rikenellaceae, Flavobacteriaceae, Victivallaceae, Coriobacteriaceae, Streptococcaceae, Porphyromonadaceae, and Ruminococcaceae [23]. Results from another study underscore the predominance of unclassified Enterobacteriaceae (21.05%), Mollicutes (4.37%), and unclassified Bacteroidaceae (2.49%) in beef cattle [24]. At the phylum level, Tenericutes (36%), Proteobacteria (30%), Fusobacteria (7.6%), and Firmicutes (1.8%) emerged as the most abundant in beef cattle [25]. Similarly, in the investigation by Deng et al. [24], Firmicutes (31.57%), Proteobacteria (24.08%), Bacteroidetes (12.96%), and Tenericutes (4.95%) constituted the predominant vaginal microbiota. It is important to mention that individual and breed differences between animals of the same species are common [4,26]. A comparative analysis of these recent studies highlights significant disparities in the proportions of predominant microbial populations among individuals.

During gestation, cows harbor a natural uterine microbiome, primarily originating from the vagina and, to a lesser extent, from the skin and gut, although less diverse than the vaginal microbiome [20,27,28]. Bacteria such as *F. necrophorum*, *Porphyromonas Levii*, and *T. pyogenes* are consistently present in the uterus during pregnancy [20] (Figure 1). Interestingly, opportunistic microbes such as *Histophilus* and *Mycoplasmataceae* have the potential to evolve into pathogenic agents under certain conditions [29,30]. These microbes, which are typically harmless in a healthy microbial ecosystem, can become pathogenic when the balance of the microbial community is disrupted or when the host’s immune system is compromised [31]. This transition can contribute to reproductive health issues and diseases in livestock. The low correlation between bacterial abundance in the uterus before calving and inflammation suggests that the uterine environment exhibits heightened microbial tolerance during gestation [32,33]. This tolerance indicates that the presence of bacteria does not necessarily lead to an inflammatory response, reflecting the uterus’s ability to manage microbial populations effectively while supporting fetal development [34]. This adaptive response is essential for maintaining a healthy pregnancy and preventing excessive inflammation [35,36].

### 2.2. Inter-Species Variation in Microbiome Composition

In the investigation of vaginal microbiomes across different cattle breeds, distinct microbial compositions have been observed. Among these, Gyr cattle, a common dairy breed prevalent in South American regions like Brazil, exhibit a notable enrichment of bacteria and fungi, with a minor presence of archaea [6]. Within the bacterial populations, Firmicutes, Bacteroidetes, Proteobacteria, and Actinobacteria are frequently identified, with *Mycosphaerella* and *Cladosporium* emerging as the predominant fungal genera. The most prevalent bacterial taxa identified in the Gyr cattle vaginal include *Aeribacillus*, *Bacillus*, *Clostridium*, *Ruminococcus*, *Bacteroides*, *Rikenella*, and *Alistipes* [6]. Notably, the *Methanobrevibacter* genus dominates among the relatively sparse archaeal population. Conversely, the vaginal microbiome of Nellore beef cattle is primarily composed of Firmicutes (40–50%), Bacteroidetes (15–25%), and Proteobacteria (5–25%), with a notable proportion of unclassified bacteria, constituting up to 20% [4]. A closer examination reveals a diverse range of bacterial genera colonizing the vaginal tract of Nellore cattle. The most abundant genera include *Aeribacillus*, *Bacteroides*, *Clostridium*, *Ruminococcus*, *Rikenella*, *Alistipes*, *Bacillus*, *Eubacterium*, and *Prevotella*, which together form a complex microbial ecosystem essential for maintaining vaginal health and potentially impacting reproductive performance [4]. In contrast, Holstein Friesian cattle, a widely distributed dairy breed found across North Africa, Europe, and the USA, display a vaginal microbiome predominantly comprised of Firmicutes, Tenericutes, Proteobacteria, and Bacteriodetes phyla [9]. Although other bacteria, such as Actinobacteria and Spirochaetae, are detected, their quantities are relatively smaller [9]. While breed differences undoubtedly play a significant role in the observed microbial compositions, management practices, and environmental factors also significantly influence the vaginal microbiome of cattle. Factors such as delivery mode, diet, housing conditions, geographic location, and veterinary care can all contribute to variations in microbial communities [26,37].

### 2.3. Temporal Dynamics across the Reproductive Cycle

In female beef cattle *Escherichia coli*, *Enterococcus faecalis*, *Yersinia enterocolitica*, *Micrococcous* sp., *Citrobacter diversus*, *Corynebacterium bovis*, *Klebsiella* sp., *Staphylococcus epidermis*, *Aerococcus vaginalis*, *Aerococcus viridans*, *Haemophilus somnus*, *Streptococcus pluranimalium*, *Sphingomonas roseiflava*, *Psychrobacter marincola*, and *Lactobacillus* spp. were detected in the vagina during a normal cycle [38,39].

Although the presence of bacteria in the cervicovaginal mucus (CVM) is not specific to uterine or vaginal inflammation, various bacteria have been detected in different parts of the genital tract [34,40,41]. In buffalo and dairy cows during the postpartum period, the presence of different bacterial types was recorded in both healthy and endometritic females. *Psychrobacter* sp. PRwf-1 and *Psychrobacter pulmonis* were predominant in both normal and endometritic buffaloes, while Tenericutes, including *Ureaplasma diversum* strain T95 and *Ureaplasma diversum* strain A417, were significantly associated with endometritis and reproductive problems in buffalo [42]. In dairy cows, during the first 50 days postpartum, *Firmicutes* are the most abundant phylum in healthy individuals, contrasting with cows suffering from uterine diseases. For example, cows with clinical endometritis show a high prevalence of *Fusobacterium* and *Trueperella*, along with a lower abundance of *Escherichia*, *Shigella*, *Lactobacillus*, *Prevotella*, *Schlegelella*, and *Streptococcus*. Over time, subclinical endometritis may persist, leading to an increased prevalence of *Anaerococcus*, *Corynebacterium*, and *Staphylococcus* [43,44]. The significant bacterial abundance in the bovine vaginal tract enables the identification of reproductive statuses, including estrous cycle, pregnancy, and metritis.

The influence of endocrine hormones on the estrous cyclicity of cows extends to the genital tract microbiome and its diversity [39]. Conversely, the genital microbiota plays a pivotal role in modulating reproductive cycle hormonal profiles [45]. Specifically, during the follicular phase, elevated estradiol levels contribute to a decrease in the pH of endometrial secretions [46], thereby shaping microbial diversity within the vagina [25]. Consequently, it appears that the microbiome present during specific phases of the estrous cycle profoundly impacts estrous cyclicity and the quality of developing oocytes [45,47].

Notably, the presence of *Lactobacillus* species is associated with a reduction in vaginal pH, fostering reproductive function by suppressing infectious pathogens, enhancing oocyte quality, and promoting luteal function [47,48,49]. Conversely, in cases of uterine infection by lipopolysaccharide (LPS)-producing bacteria like *E. coli*, an inflammatory cascade is triggered in the endometrial tissue, leading to the accumulation of LPS in the antrum [50,51]. Moreover, granulosa cells possess specific Toll-like receptors 4 (TLR-4) receptors for LPS recognition, eliciting an inflammatory response within the follicular cells. This response compromises steroidogenic activity and oocyte development by inhibiting mitotic activity [52,53].

Clinical investigations have corroborated these findings, linking severe uterine contamination to reduced follicle and corpora lutea sizes, ultimately resulting in diminished peripheral plasma concentrations of estradiol and progesterone [49,54]. Such disruptions in hormonal profiles are likely contributors to subfertility in cattle.

Studies have detected the presence of aerobic bacteria such as *Escherichia coli*, *Bacillus* sp., *Staphylococcus* spp., *Streptococcus* spp., and *Proteus* sp. in the vagina of healthy cattle [55]. Additionally, Deng et al. [24] reported an abundance of *Histophilus*, *Clostridium*, and *Campylobacter* in the vagina during the first trimester compared to pre-breeding. Symbiotic bacteria present in the genital tract during pregnancy may have beneficial effects. The absence of *Lactobacilli* in CVM of women during pregnancy was associated with increased secretion of IL-8 and premature delivery [56].

In pregnant buffaloes, bacteria such as *Escherichia coli*, *Klebsiella* sp., *Staphylococcus* sp., and *Bacillus* sp. are commonly isolated from the genital tract [57]. The normal uterine microbiome of Nili-Ravi buffaloes during pregnancy predominantly consists of *Escherichia coli*, *Staphylococcus* sp., *Lactobacillus* sp., *Proteus* sp., and *Micrococcus* sp. [58]. In contrast, *Citrobacter* sp. is more frequently found in buffaloes that have been aborted, suggesting its potential as an indicator of premature delivery or abortion [58]. Similarly, the normal microbiome of Murrah buffaloes includes Gram-positive bacteria such as *Staphylococcus* sp., *Streptococcus* sp., and *Bacillus* sp., alongside Gram-negative bacteria like *Escherichia coli*, *Proteus* sp., and *Klebsiella* sp. [59].

**Figure 2:**
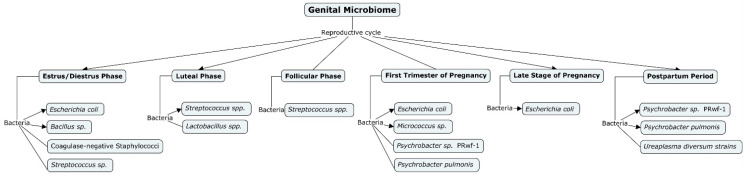
Microbial diversity in bovine reproductive cycle. This conceptual map categorizes the bacteria found in the bovine reproductive system according to reproductive cycle stages. It outlines the different bacterial taxa present during normal reproductive phases (estrus, diestrus, luteal, follicular) and various stages of pregnancy (first trimester, late stage, postpartum).

Regarding pregnancy stages, the vaginal microbiome appears to be more diverse in the first stage of gestation compared to the late stage, with *Escherichia coli* (27.08%) and *Micrococcus* sp. (20%) being predominantly isolated throughout gestation in buffalo [38] (Figure 2). NGS studies have shown reductions in diversity and richness of microbial species during pregnancy, with *Lactobacillus iners*, *Lactobacillus crispatus*, *Lactobacillus jensenii*, *Lactobacillus johnsonii*, *Lactobacillales*, *Clostridiales*, *Bacteroidales*, and *Actinomycetales* being the most abundant [60]. This highlights the need for similar investigations in cows, as it is highly possible that low microbial diversity in cows could lead to abortion and reproductive disorders [35]. Further research is necessary to establish the relationship between microbial diversity and reproductive outcomes in cattle.

To analyze disturbances in vaginal microbial communities, a normal CVM status should be defined, distinguishing between normal and pathological microorganisms. The bacterial signature should be defined according to every period of the estrous cycle, pregnancy, and postpartum. Recent studies have developed random forest methods to detect pregnancy status based on vaginal and fecal microbiomes [24]. Despite these advancements, significant variations in vaginal microbiome diversity between gestational stages have been reported. Further investigations of vaginal microbiomes during pregnancy and the evaluation of factors affecting the diversity and dynamics of the vaginal community in long-term studies are warranted.

## 3. Microbial Functions and Mechanisms

### 3.1. Microbial Functions (Beyond Passive Commensals)

Communication through odor-based signals is a fundamental aspect of inter-animal communication across various species [61,62,63]. Particularly in animal reproduction, the excretion of specific semiochemical substances plays a pivotal role in attracting males for copulation [64,65,66]. Recently, there has been a growing interest in the role of bacterial odor production in animals, particularly in the context of producing pheromone signals for communication between conspecifics [62,67,68]. The microbiota can directly influence chemical signals, thereby influencing a variety of the host’s social behaviors, including sexual signaling [69,70] (Table 1).

In wild meerkats, for example, social odors have been observed to vary based on the bacterial community present in anal secretions [71]. The microbial metabolism within the host alters the volatile fatty acid profiles from scent glands, resulting in the production of sex-specific odors that influence chemical communication in mammals [72]. A proposed origin of axillary malodor pathways suggests that aliphatic amino acids are metabolized into short-chain (C2–C5) volatile fatty acids (VFAs) by *Staphylococcus* [72].

Furthermore, the relationship between the host microbiome and the fermentation hypothesis for chemical recognition posits that variations in pheromone signals produced by mammalian scent glands are mainly due to discrepancies in the abundance of specific bacterial communities [72,73]. Such mechanisms highlight the intricate interplay between microbial communities and chemical signaling in animal communication.

### 3.2. Immune Modulation and Pathogen Defense

The genital microbiome plays multifaceted roles in immune modulation and pathogen defense, with key mechanisms including the aggregation of pathogens to impede their adherence to host tissues. For example, *Lactobacillus rhamnosus* GG has been demonstrated to bind to the surface of *Escherichia coli* in vitro, inhibiting its adhesion to intestinal epithelial cells, suggesting the potential utility of the genital microbiome in combating pathogenic microorganisms [74].

In addition to pathogen aggregation, the genital microbiome exerts immune-modulating effects through diverse mechanisms, impacting immune cells, cytokine production, and the secretion of bioactive molecules. Strains of *Lactobacilli* and *Bifidobacteria* influence immune function by regulating various components of the immune system, including enterocytes, antigen-presenting cells, regulatory T cells, and effector T and B cells [75]. Furthermore, the genital microbiome produces bioactive molecules such as short-chain fatty acids (SCFAs), hydrogen peroxide, and bacteriocins, which exhibit antimicrobial and anti-inflammatory properties [76]. *Lactobacilli* metabolizes dietary carbohydrates through glycolysis to produce lactic acid. This process reduces the pH of the environment, creating unfavorable conditions for the growth of pH-intolerant pathogens [77]. In addition to lactic acid, SCFAs such as acetic acid are produced through the fermentation of polysaccharides and oligosaccharides [78] (Table 1). These substrates are typically derived from dietary fibers and glycogen. The main pathways involved in SCFA production include glycolysis and fermentation, where *Lactobacilli* convert glucose and other hexose sugars into pyruvate via glycolysis or lactate dehydrogenase. Pyruvate is then fermented to produce lactic acid and other SCFAs.

Moreover, the genital microbiome stimulates the immune system by promoting cytokine production and the release of immune factors. The degradation of bacterial components responsible for *Lactobacilli* adhesion results in antimicrobial peptide production, enhancing host defense [79]. Bacteriocin-mediated killing commonly involves the destruction of target cells through pore formation or inhibition of cell wall synthesis [80]. For instance, *Bifidobacterium bifidum* NCFB 1454 produces Bifidocin B, which exhibits activity against Gram-positive bacteria [81]. Additionally, the genital microbiome induces the secretion of defensins from epithelial cells, providing antimicrobial activity against various pathogens [82]. *Lactobacillus* strains also synthesize antifungal compounds such as benzoic acid, methylhydantoin, and mevalonolactone [83,84]. For instance, benzoic acid is synthesized through the shikimate pathway, which involves the conversion of simple carbohydrates into aromatic amino acids, which are then decarboxylated to produce benzoic acid [85]. The biosynthesis of methylhydantoin involves the hydrolysis and cyclization of amino acids like arginine through enzymatic reactions, often involving urease and other hydrolase enzymes. Mevalonolactone is produced via the mevalonate pathway, which is crucial for the synthesis of isoprenoids. The pathway begins with acetyl-CoA, which is converted to mevalonate through a series of enzyme-mediated steps, including the action of HMG-CoA reductase [86]. Specifically, *Lactobacillus plantarum* FST 1.7, isolated from wheat grains, produces four antifungal substances, including lactic acid, phenyllactic acid, and two cyclic dipeptides (cyclo(L-Leu-L-Pro) and cyclo(L-Phe-L-Pro)) [87].

During calving, the cervix dilates, allowing bacteria from the external environment to enter the vagina and subsequently contaminate the uterus. In cows that develop endometritis, the bacteria present in the uterus postpartum may act as either etiological agents or secondary infections [34]. Molecular-based methods targeting the 16S rRNA gene in vaginal and uterine samples have revealed a high prevalence of pathogenic bacteria such as *Fusobacterium* and *Corynebacterium*, which are commonly associated with metritis, endometritis, and infertility [43,44]. However, the presence of other bacterial species is also crucial in regulating endometrial inflammation. For example, *Lactobacillus* has been shown to interfere with the secretion of proinflammatory cytokines stimulated by *E. coli* [88].

In vivo studies have demonstrated that combining *Lactobacillus* and *Pediococcus* cultures results in robust control of the endometrial inflammatory response induced by *E. coli* [89]. Additionally, *Lactobacillus* species produce bioactive molecules such as lactic acid and hydrogen peroxide, which inhibit the growth of pathogens like *Staphylococcus aureus* and *Trueperella pyogenes*, commonly isolated from cattle suffering from uterine diseases [21,90]. Lactic acid, in particular, is a potent acidic substance capable of penetrating sensitive microbes without specific receptors, thereby increasing cytosol acidity and leading to bacterial death [91].

## 4. Factors Influencing Microbiome Dynamics

### 4.1. Dietary Impact on Microbiome Composition

The microbial composition of the uterus in dairy cows during the postpartum has been shown to be influenced by dietary factors, particularly the energy content, in the vicinity of calving [37].

A negative restricted diet in dairy cattle results in higher levels of non-esterified fatty acids (NEFA) and beta-hydroxybutyrate (BHB), along with lower total cholesterol (TC) concentrations [37]. These changes reflect an altered energy balance that significantly impacts both the immune system and the microbiome. At the immune level, negative energy balance upregulates proinflammatory molecules such as serum amyloid A3 (SAA3), CXC chemokine receptor 2 (CXCR2), Interleukin (IL) 1 and IL8, IL6, and lipopolysaccharide-binding protein (LBP). This upregulation signifies a heightened uterine inflammation during periods of energy deficit [37]. At the microbiome level, negative energy balance is associated with specific shifts in microbial composition, compared to females received 100% on energy requirements. During the first month postpartum, Bacteroidetes and Fusobacteria are the most abundant phyla, while Proteobacteria are the least abundant. These changes indicate that the immune response influenced by the energy balance also affects the uterine microbiota composition. A comparative analysis of the uterine microbiome pre-calving and post-calving in cows that developed metritis versus those that did not reveal distinct differences. Cows experiencing metritis displayed a higher prevalence of Bacteroides and Fusobacteria, coupled with a reduced abundance of Proteobacteria and Tenericutes, in stark contrast to their non-metritic counterparts [35].

These findings underscore the profound impact of nutrition on the genital microbiome, mediated through the modulation of overall metabolism and immune functions, consequently influencing the occurrence of dysbiosis and genital infections.

### 4.2. Environmental Factors and Microbial Resilience

From an evolutionary perspective, it has been proposed that the vaginal microbiome could have originated from the intestinal microbiota. This hypothesis is supported by marked similarities observed between the microbial populations of these two anatomical parts [4]. The proximity of the vagina and anus, along with the frequent contact between feces and the vulva, suggests a potential route for microbial transmission [4]. However, current prevailing thoughts suggest an alternative origin for the genital microbiome of neonates, which initially derives from maternal tissues in contact with the neonate after parturition [26,92].

Subsequently, the genital microbiome undergoes numerous changes throughout the lifetime of a female, influenced by various factors, including contamination from nearby organs such as the gastrointestinal tract. Recent research findings lend support to this hypothesis [24]. When comparing changes in microbial populations in fecal and vaginal samples collected before mating and at different stages of the gestational period, it was observed that fecal microbial diversity remained consistent, while the vaginal microbiome exhibited dynamic changes across various gestational stages.

## 5. Microbiome and Reproductive Health

### 5.1. Microbial Impact on Fertility and Reproductive Outcomes

The microbiome present in the genital tract during oocyte development is believed to significantly influence normal sperm functions and fertilization capabilities, which is associated with reduced conception rates and prolonged calving intervals in cattle [47] (Table 2). The postpartum period represents a crucial phase in the reproductive cycle of cattle, where successful uterine involution is essential for optimal fertility [93]. Disruptions in this process can lead to conditions such as metritis, endometritis, or cystic ovarian disease, which negatively impact reproductive performance [94]. Ideally, uterine involution should proceed without interruption and be completed by approximately 45 days postpartum [13]. However, bacterial contamination of the uterus during parturition poses a significant risk, potentially delaying involution and resumption of ovarian activity and thereby prolonging calving-first insemination and calving-fertilizing insemination [95,96,97].

Sperm, akin to bacteria, are recognized as foreign bodies by the immune system of the genital tract, prompting an inflammatory response upon attachment to Toll-like receptors (TLRs). Notably, TLR-2, located at the endometrial glands, plays a pivotal role in eliminating “less fit” and excess spermatozoa, potentially preventing polyspermy and preparing the uterus for implantation [98]. Postpartum, uterine infections disrupt endometrial epithelial integrity and reduce uterine gland functions, compromising folliculogenesis and fertility [48,99]. Metritis, for instance, can extend the duration of days open, intercalving intervals, and the number of services per conception, leading to increased involuntary culling within the herd and, consequently higher economic losses [100,101]. Several reproductive infections, including those involving *Escherichia coli*, *Trueperella pyogenes* exotoxin, and *Bovine Herpesvirus 4* (BoHV-4), are known to disrupt reproductive hormone secretion [50,102,103].

For example, the onset of metritis is linked to the presence of *Escherichia coli* and its specific virulence factors in the uterus during the initial days postpartum [104]. *Escherichia coli* releases lipopolysaccharide (LPS), which elevates prostaglandin E2 (PGE2) production by the endometrial glands instead of PGF2α. While PGF2α is a luteolytic hormone, PGE2 functions as a luteotropic hormone, leading to the persistence of the corpus luteum in the absence of conception, resulting in pseudopregnancy, luteal cysts, anestrus, and increased calving intervals [48,50] (Table 2). Additionally, progesterone produced by the persistent corpus luteum dampens immune responses, facilitating pathogen proliferation and impairing fertility [105,106]. Highly pathogenic bacteria like *Trueperella pyogenes* produce aggressive toxins such as pyolysin, damaging endometrial epithelial cells and disrupting hormonal secretion, contributing to decreased conception rates and increased postpartum anestrus [34]. Furthermore, low counts of certain bacteria like *Corynebacterium*, *Staphylococcus*, and *Prevotella* two days before insemination have been linked to improved pregnancy rates in cattle [107].

While the beneficial effects of intravaginal *Lactobacillus* are not yet fully understood, in the gut, it produces phenolics that protect the oocyte against oxidative stress, thereby enhancing oocyte competency and fertilization [108,109,110]. Infusing *Lactobacillus* as probiotics during cattle breeding could potentially improve fertility by promoting the ovulation and fertilization of better-quality oocytes with increased conception rates and reduced services per conception in cattle [108,111]. Additionally, *Lactobacillus* suppresses the production of eicosanoids, which are implicated in inflammatory reactions and can negatively affect oocyte quality when produced at high concentrations. In humans, *Lactobacillus delbrueckii*, abundant in the vagina, increases sperm capacitation through the production of bicarbonate ions (HCO_3−_) from water and carbon dioxide [112,113]. Further studies are needed to explore the benefits of using such probiotics around the time of breeding in cattle, which may lead to reduced services per conception, increased conception rates, and higher pregnancy rates.

Fluorescence in situ hybridization (FISH) and 16S rRNA gene sequencing have allowed for the identification of bacterial species in pregnant cattle [20,27]. Contrary to previous assumptions, it has been revealed that the gravid uterus is not sterile, with the microbiomes differing widely from those found in diseased endometria [114]. The abundance and diversity of microbiomes within the genital tract during pregnancy are relatively low, likely to reduce the risk of dysbiosis and abortion [4,24]. Common bacterial phyla found in the pregnant uterus of cattle include Firmicutes and Bacteroidetes [27]. Additionally, studies have detected an abundance of certain bacteria like *Fusobacterium necrophorum*, *Trueperella pyogenes*, and *Porphyromonas levii* in the endometrium and placentomes of cows [20]. While the *Lactobacillus* species are not commonly detected in the bovine endometrium, the presence of beneficial *Lactobacillus* species in the endometrium has been associated with improved pregnancy rates and reduced incidence of abortion, highlighting the importance of microbial balance in reproductive health [115,116,117] (Table 2). Conversely, dysbiosis of the vaginal and endometrial microbiomes, characterized by an increased abundance of pathogenic bacteria, has been linked to fertility issues, emphasizing the importance of microbial balance in reproductive health [55,118].

### 5.2. Microbial Factors in Reproductive Disorders

The postpartum is a critical phase in the cattle production cycle, during which uninterrupted uterine involution is vital to prevent complications like metritis, endometritis, or cystic ovarian disease. Optimal uterine involution should ideally be completed around 45 days postpartum, but bacterial contamination during parturition can impede this process [95,96,97]. These bacteria originate from the external environment or the animal’s adjacent organs [27,119]. Retained fetal membranes (RFM), metritis, and endometritis are the most common postpartum complications in cattle. In cows with normal pregnancy and parturition, all females initially harbor a similar microbiome at calving, which diverges significantly by seven days postpartum (DPP), especially in cows that experienced dystocia or RFM, where Fusobacteria and Bacteroidetes become predominant [97]. The genital tract microbiome during postpartum is believed to profoundly influence subsequent reproductive performances [22,120]. RFM, characterized by the persistence of placental tissue attached to the endometrium, creates a favorable environment for the growth of pathogenic bacteria, thereby leading to subfertility [121]. Culture- and molecular-based microbiology identification methods have linked RFM predominantly to *Escherichia coli* (68%) and *Staphylococcus aureus* (18%) [122], while also highlighting RFM as a significant risk factor for uterine diseases such as metritis and endometritis [121,123,124] (Table 3).

Metritis, characterized by deep inflammation of the endometrium and myometrium, typically manifests with purulent or fetid vaginal discharge within the first 21 days postpartum [125]. Cows with metritis tend to harbor a less diverse microbiome, often dominated by *Bacteroides*, *Porphyromonas*, and *Fusobacterium* [35,118]. There is evidence suggesting that a higher abundance of Proteobacteria in the vagina seven days before calving predicts the occurrence of postpartum metritis, possibly through synergistic interactions with Fusobacteria [97] (Table 3). Notably, *Escherichia coli* is frequently detected in uterine samples from cows with metritis, with the severity of symptoms often linked to strains harboring the virulence factor kpsMTII [126]. Additionally, a symbiotic relationship between *Escherichia coli*, *Trueperella pyogenes*, and *Fusobacterium necrophorum* facilitates colonization of the endometrium and evasion of the immune system, contributing to metritis [32]. *Trueperella pyogenes* produces pyolysin, a potent cytolytic substance that damages endometrial epithelial cells, disrupts tissue integrity, and triggers inflammatory reactions [34]. While endometrial cells do not respond to damaged-associated molecular patterns (DAMP), the combination of pathogens and DAMP leads to intracellular secretion of IL-1, initiating an inflammatory cascade [34].

Endometritis, characterized by superficial inflammation of the endometrium, typically presents with milder clinical symptoms compared to metritis and is detected after 21 days postpartum, reflecting abnormally sustained postpartum inflammation [94,125]. The origin and pathogenesis of endometritis are subjects of debate, with microbial isolation reported in animals with clinical and subclinical endometritis [34,120]. Metagenomic analyses of uterine microbiomes in endometrial cytobrush samples collected during the first week postpartum have revealed that metritis and clinical endometritis are associated with lower microbial diversity, dominated by *Bacteroides*, *Fusobacterium*, and *Trueperella*, and reduced abundance of *Escherichia*, *Shigella*, *Lactobacillus*, *Prevotella*, *Schlegelella*, and *Streptococcus* [34,43,97] (Table 3). Additionally, higher levels of *Anaerococcus*, *Corynebacterium*, and *Staphylococcus* increase the risk of subclinical endometritis in postpartum cows [34,44,97].

A significant variation was observed among individuals in the uterine microbiome of cows without uterine infections during the first month postpartum [43,44]. Despite this variation, alpha and beta diversities were consistent across different postpartum days (10, 21, and 35 DPP), indicating similar bacterial diversity in the uterus of cows without uterine inflammation, irrespective of the sampling time [43]. However, notable differences were observed in the uterine microbiome between cows with and without uterine inflammation [43]. Metagenomic analysis of uterine samples collected at multiple time points during the first 35 DPP revealed that the uterine microbiome of cows without inflammation predominantly consisted of *Porphyromonas*, *Bacillus*, *Schlegelella*, *Paracoccus*, and *Fusobacterium* [43].

Of particular interest, the vaginal and uterine microbiomes of cows without uterine inflammation during the first 50 DPP exhibited remarkable similarity and were both highly enriched with *Firmicutes* [44]. This similarity may be attributed to the relatively unconstructed cervical lumen soon after calving, facilitating the mixing of vaginal and uterine contents and their movement throughout the reproductive tract. It is hypothesized that in cows without uterine inflammation, the genital microbiome remains uncontaminated during calving by external microbes, or at least remains unaffected for an extended period postpartum. A retrospective comparison of the genital microbiome of pregnant cows before calving with those developing endometritis after 21 DPP, and those maintaining an uninfected uterus, revealed that the genital microbiome pre-calving resembled the vaginal microbiome of healthy cows that did not develop uterine infections beyond 21 DPP [44].

It is noteworthy that the decrease in vaginal microbiome quantity affects sexual attractiveness in estrus ewes [127]. Behavioral observations of female hamsters indicate that vaginal secretion could play a role in olfactory communication, and changes in their qualitative and quantitative profiles of volatile molecules would affect sexual behavior [128]. Microbes play an essential role in odor production in general; particularly, they influence hamster vaginal secretion for odor production. The vaginal pheromone copulins are reported to be produced under the influence of bacteria in rhesus monkeys [129,130].

In female baboons, the richness of lactic acid-producing bacteria varies during various phases of the ovarian cycle, with major shifts in bacterial composition occurring during the ovulatory phase [131,132]. Detecting *Simonsiella* sp. in the vagina helps identify estrus in bitches [133]. Future studies on changes in the vaginal microbiome at different stages of the estrous cycle and its association with follicular dynamics and steroid profiles would be of valuable interest in bovine reproductive management.

## 6. The Role of the Male Reproductive Microbiome in Fertility

### 6.1. Microbial Diversity in the Male Reproductive Tract

The male reproductive microbiome significantly influences fertility in bovine species, affecting sperm quality, semen characteristics, and overall reproductive health [134,135]. Research has underscored the diverse microbial communities present in the bovine male reproductive tract, including the testes, epididymis, and ejaculate, and their potential impact on reproductive outcomes [33,136].

Conducting 16S rRNA gene sequencing has revealed that the seminal microbiome in bulls is predominantly composed of facultative anaerobic and strictly anaerobic microorganisms. Research has shown that bacterial loads differ substantially across cattle breeds, with Jersey bulls exhibiting the highest levels, followed by crossbred cattle. In comparison, Zebu breeds, such as Gir and Red Sindhi, tend to have lower bacterial loads [137]. Key bacterial phyla identified in bull semen include Actinobacteria, Bacteroidetes, Euryarchaeota, Firmicutes, Fusobacteria, and Proteobacteria [134]. Other studies have consistently identified Firmicutes, Proteobacteria, Fusobacteria, Actinobacteria, and Bacteroidetes as the dominant phyla present in bull seminal microbiomes [138,139].

These microbial communities can vary with factors such as age, health status, and environmental conditions [135]. For instance, negative correlations have been observed between specific bacterial populations and several sperm quality parameters. These include sperm motility [140], viability, membrane integrity, and acrosome reaction [141], as well as increased rates of sperm DNA fragmentation [142] and reductions in the total sperm count [143].

### 6.2. Clinical Implications and Management

The role of the male reproductive microbiome has significant implications for reproductive management in bovines. Probiotic interventions, akin to those used in female reproductive health, have shown promise in improving semen quality and fertility outcomes [144,145]. In particular, *Lactobacillus* has been found to enhance sperm quality, fertility and fertility [145,146]. Conversely, these bacteria can transmit diseases to females during natural mating, potentially hindering fertilization and subsequently reducing pregnancy rates [138,147,148]. These findings suggest that managing the male reproductive microbiome through dietary and probiotic strategies could be a viable approach to improving reproductive efficiency in bovine herds.

## 7. Microbiome-Based Interventions for Disease Management

### 7.1. Utilizing Microbiome as Probiotics in Livestock Disease Management

Managing reproductive diseases in cattle presents a significant challenge for both veterinarians and farmers. Despite antibiotic treatment for metritis, only 67 to 77% of cases recover from clinical symptoms, with fertility remaining compromised [149,150]. In human medicine, modulating the genital microbiome with probiotics has emerged as an effective strategy [151,152,153]. For instance, intravaginal administration of lactic acid bacteria has been shown to modify the uterine microbiome and prevent conditions such as recurrent vulvovaginal candidiasis [151].

Lactic acid bacteria (LAB) are a diverse group of Gram-positive bacteria characterized by their production of lactic acid as the primary metabolite during carbohydrate fermentation, making them highly tolerant to acidic environments. These bacteria belong to various taxa, predominantly within the order Lactobacillales of the phylum Firmicutes, as previously discussed. The most commonly referenced genera of LAB include *Lactobacillus*, *Pediococcus*, *Leuconostoc*, and *Weissella* (collectively known as LPLW), which are closely related phylogenetically and are often categorized together as the *Lactobacillus* group [154,155]. In cattle, bacterial isolates from vaginal samples have been explored for their potential as probiotic candidates to prevent or treat uterine infections. The use of these probiotic strains has been proposed as an alternative strategy for managing postpartum uterine infections and reducing inflammation [156]. Notably, research by Pellegrino et al. [157] demonstrated that LAB strains isolated from the vaginal tract exhibit a strong capacity to produce hydrogen peroxide (H_2_O_2_), a key factor in their antimicrobial activity.

In cattle, intravaginal treatment with a blend of lactic acid bacteria, including *Lactobacillus rhamnosus* CECT 278, *Pediococcus acidilactici* CECT 5915, and *Lactobacillus reuteri* DSM 20016, three weeks before calving reduced metritis prevalence by 58% [153]. Likewise, administering vaginal probiotic treatment during the three weeks before calving has been linked to a reduced incidence of metritis [158]. Probiotic treatment with lactic acid bacteria has been found to modulate the inflammatory response by downregulating the expression of mRNA transcripts encoding for specific proteins, such as L-selectin. This protein is involved in neutrophil infiltration into infected tissues and the expression of genes related to degranulation and phagocytosis [13,153,159] (Table 4).

The observed reduction in neutrophil activity may be attributed to decreased levels of pathogenic bacteria in the genital tract, either through competition with lactic acid bacteria or by coaggregation with more pathogenic microbes, thereby reducing the adhesion of pathogens to specific cell surface receptors [74,153]. In vivo and ex vivo studies have demonstrated that the combination of *Lactobacillus rhamnosus*, *Pediococcus acidilactici*, and *Lactobacillus reuteri* leads to a significant reduction in the inflammatory response of endometrial epithelial cells when challenged with *Escherichia coli* [89] (Table 5). This suggests that administering these probiotics may help mitigate the inflammatory effects of pathogenic bacteria, potentially improving uterine health in dairy cattle. Additionally, in vitro experiments have shown that the growth of *Staphylococcus aureus*, a major pathogen associated with postpartum infections in dairy cattle, is inhibited by treatment with *Lactobacillus gasseri* strains CRL1421 and CRL1412 [90] (Table 4). These findings suggest that probiotics could be a valuable tool in managing uterine diseases and promoting reproductive health in dairy cattle by reducing inflammation and inhibiting the growth of harmful bacteria [160].

**Table 4:** Probiotic strains and their effects on cattle reproductive health. It outlines various probiotic strains used in cattle, their methods of administration, targeted diseases, observed health benefits, and key references. It highlights the role of specific probiotics in improving reproductive health outcomes, such as reducing metritis prevalence and enhancing fertility metrics.

Probiotic Strain	Method of Administration	Disease Targeted	Observed Effects	Key References
*Lactobacillus rhamnosus*, *Pediococcus acidilactici*, *Lactobacillus reuteri*	Intravaginal	Metritis	Reduced metritis prevalence by 58%, modulation of inflammatory response	[153]
*Lactobacillus sakei*, *Pediococcus acidilactici*	Intravaginal	Uterine diseases	Modulation of immune reactions, reduced uterine inflammation	[156]
*Lactobacillus sakei* FUA3089, *Pediococcus acidilactici* FUA3138, FUA3140	Intravaginal	Reproductive performance	Improved productive and reproductive performances, reduced uterine inflammation	[76]
*Lactobacillus buchneri*	Intravaginal	Postpartum uterine health	Improved uterine health, higher conception rates, shorter median days to conception	[161]
*Lactobacillus gasseri* CRL1421, CRL1412	In vitro	Postpartum infections	Inhibited growth of *Staphylococcus aureus*	[21]

**Table 5:** Mechanisms of probiotic action in reproductive health. It details the mechanisms by which probiotics exert their beneficial effects on reproductive health in cattle, providing insights into how probiotics contribute to improved uterine health and fertility.

Mechanism	Description	References
Competition	Probiotics outcompete pathogenic bacteria for adhesion sites	[153]
Coaggregation	Probiotics coaggregate with pathogens, reducing their adhesion to cell receptors	[74]
Anti-inflammatory effects	Downregulation of mRNA transcripts for inflammatory proteins, reducing neutrophil infiltration	[153,159]
Inhibition of pathogenic growth	Probiotics produce substances like Pediocin that inhibit growth of pathogens	[162,163]

### 7.2. Disease Prevention and Treatment Strategies

A variety of probiotics are commonly utilized in livestock, particularly in cattle management. These probiotics include various strains of LAB, such as *Lactobacillus bulgaricus*, *Lactobacillus buchneri*, *Lactobacillus acidophilus*, *Lactobacillus casei*, *Lactobacillus lactis*, *Lactobacillus salivarius*, and *Lactobacillus plantarum*, alongside other beneficial microorganisms like *Streptococcus thermophilus*, *Enterococcus faecium*, *Enterococcus faecalis*, and different *Bifidobacterium* species [76,156,161,164,165]. Additionally, organisms such as *Aspergillus oryzae* and yeast strains like *Saccharomyces cerevisiae* find application in this context [165]. Among the key probiotic strains studied for promoting uterine health in cattle are various *Lactobacillus* and *Bifidobacterium* species. These probiotics are administered through various means, including intravaginal pessaries, intrauterine infusions, and oral supplements. Recent studies have highlighted the protective role of *Lactobacillus* strain SQ0048, which is present in the vaginal microbiota of healthy cows. This strain acts as a significant barrier against genital pathogens by adhering to the specific epithelial surfaces and producing inhibitory substances [166]. Probiotic strains, including LAB, exert their effects on pathogenic microorganisms through various mechanisms, such as enhancing intestinal barrier function, increasing mucin production, and modulating immune system activity [167].

When it comes to LAB strains, their effectiveness can be categorized based on their adherence to the endometrium (Table 6). This ranges from low adherence with strains like *Lactobacillus sakei* and *Lactobacillus reuteri* to moderate to strong adherence with strains like *Pediococcus acidilactici* and *Lactobacillus rhamnosus*, which are adept at forming biofilms [168,169]. Moreover, certain combinations of LAB species, when used as probiotic mixtures, have shown superior abilities in modulating inflammation compared to individual strains. For instance, a specific blend of *Lactobacillus rhamnosus*, *Lactobacillus acidilactici*, and *Lactobacillus reuteri* exhibited robust anti-inflammatory effects on endometrial epithelial cells, resulting in reduced *Escherichia coli* infection both in vitro and ex vivo [89,168]. This blend notably downregulated the expression and secretion of key inflammatory molecules like chemokines and cytokines induced by *Escherichia coli* [89].

An in vivo study investigating the impact of intravaginal infusion of a probiotic mixture composed of *Lactobacillus sakei* FUA3089, *Pediococcus acidilactici* FUA3138, and *Pediococcus acidilactici* FUA3140 reported significant modulation of immune reactions and reduced incidence of uterine diseases following treatment [156]. Intravaginal administration of LAB has shown efficacy in reducing the occurrence of metritis in dairy cows compared to control groups [156]. Additionally, a combination of LAB strains, including *Lactobacillus sakei* FUA 3089, *Pediococcus acidilactici* FUA 3140, and *Pediococcus acidilactici* FUA 3138, has been shown to significantly improve both productive and reproductive performances while reducing uterine inflammation [76]. Therefore, probiotics, encompassing strains such as *Lactobacillus acidophilus*, *Lactobacillus rhamnosus*, and *Eenterococcus faecium*, emerge as effective tools for managing reproductive diseases in cattle, offering promising prospects for enhancing overall herd health and productivity.

The direct intravaginal infusion of *Lactobacillus* species as probiotics holds significant promise for mitigating postpartum uterine diseases in cattle. Studies have demonstrated a notable reduction in incidences of endometrial inflammation following intravaginal introduction of *Lactobacillus sakei* and *Pediococcus acidilactici* both before and after calving [76,156]. *Pediococcus* species, in particular, offer valuable probiotic properties, as they harbor specific genes encoding for Pediocin, a potent bactericidal peptide [162,163].

Moreover, intravaginal administration of *Lactobacillus buchneri* in dairy cows between 24 and 30 days postpartum resulted in notable improvements in uterine health status and reproductive performances compared to control groups treated with isotonic saline solution [161]. The probiotic-treated group exhibited shorter median days to first service, a reduced number of services per conception, higher first-service conception rates, and shorter median days to conception. Furthermore, the expression of pro-inflammatory cytokines and chemokines was notably lower in the probiotic-treated group. These findings underscore the protective role of *Lactobacillus* species in maintaining genital health and enhancing fertility in cattle, emphasizing the potential of probiotic interventions as effective management strategies for postpartum uterine diseases [160].

## 8. Advanced Techniques in Microbiome Analysis

### 8.1. Methodologies for Data Collection and Analysis

To accurately study the reproductive microbiome, robust and reproducible methodologies are required. Common sampling techniques include swabbing of the vaginal canal or cervix, uterine flushing, and, in some cases, fecal sampling to explore the gut-reproductive microbiome axis [170,171]. Samples are typically preserved in nucleic acid stabilization buffers and stored at −80 °C to prevent DNA degradation [171]. For culture-dependent studies, samples are immediately inoculated on appropriate growth media, such as blood agar, MacConkey agar, or specialized anaerobic media [172]. Culture results are often coupled with molecular methods like MALDI-TOF MS (Matrix-Assisted Laser Desorption/Ionization–Time of Flight Mass Spectrometry) for rapid bacterial identification [172]. In molecular studies, DNA extraction is generally performed using commercial kits such as the Qiagen DNeasy or MoBio PowerSoil kits, depending on the sample type [173] (Table 7). Sequencing data are processed using bioinformatics tools such as QIIME 2 and DADA2, which facilitate sequence filtering, denoising, and taxonomic classification [171].

### 8.2. Culture-Dependent Techniques

Culture-dependent techniques allow for the study of microbial physiology, antibiotic resistance, and pathogenicity, which are not always evident through culture-independent methods like NGS [174]. For example, specific bacterial species associated with reproductive diseases, such as *Escherichia coli*, *Trueperella pyogenes*, and *Fusobacterium necrophorum*, have been successfully cultured and tested for their virulence and resistance profiles using traditional microbiological techniques [175,176].

However, culture-dependent methods have limitations, as many reproductive tract microbes are anaerobic, fastidious, or exist in a viable but non-culturable (VBNC) state [134]. As a result, only a fraction of the microbial diversity present in the reproductive tract can be captured through cultivation (Table 7). To address this, researchers often combine culture-dependent and culture-independent methods to gain a more comprehensive understanding of the reproductive microbiome [177].

### 8.3. High-Throughput Sequencing for Microbiome Studies

High-throughput sequencing has revolutionized microbiome studies in cattle by enabling comprehensive and detailed analysis of microbial communities. This technique allows researchers to sequence millions of DNA fragments simultaneously, providing a deep insight into the composition and dynamics of the cattle microbiome.

Researchers have employed 16S rRNA gene sequencing to characterize the microbial diversity and abundance in the organs of different species [136,178] (Table 7). In reproductive health studies, high-throughput sequencing has been instrumental in elucidating the role of the genital microbiome in conditions such as metritis and endometritis in cows. By analyzing the microbial composition and gene expression profiles, researchers have identified potential biomarkers and pathways associated with uterine health and disease [28,179].

Furthermore, high-throughput sequencing has facilitated longitudinal studies to monitor microbiome dynamics throughout different stages of cattle development, reproduction, and disease progression. This longitudinal approach provides valuable insights into temporal changes in microbial communities and their correlation with physiological parameters and health outcomes [180]. This latter study investigates the microbiota in the udder environment of cows, which was previously thought to be sterile. Using high-throughput sequencing, researchers followed the microbiota dynamics over 5 months in 10 cows, categorized by low and high somatic cell counts (SCC). They found that cows with low SCC had a dynamic and diverse microbiota that could quickly recover from imbalances. In contrast, cows with high SCC maintained a consistent dominance of certain genera, often associated with higher SCC or infections. The findings suggest that the diversity and composition of udder microbiota are linked to udder health and SCC levels, which has significant implications for managing and treating mastitis.

These observations could also be valid for uterine and vaginal environments due to the similar principles of microbial dynamics and health correlations. Like the udder, the uterine and vaginal microbiomes can influence and be influenced by health status, reproductive stages, and infections. A diverse and balanced microbiome may enhance resilience against pathogenic invasions and promote reproductive health, while a less diverse microbiome could be associated with higher risks of infections and reproductive disorders.

### 8.4. Functional Metagenomics and Omics Approaches

Functional metagenomics and omics approaches have emerged as powerful tools for studying the microbiome of cattle and its functional contributions to various physiological processes [181]. By employing techniques such as metagenomics, metatranscriptomics, metaproteomics, and metabolomics, researchers can gain comprehensive insights into the functional potential, gene expression patterns, protein profiles, and metabolite compositions of the cattle microbiome [182,183] (Table 7). These approaches enable the identification of microbial genes involved in key metabolic pathways, host–microbe interactions, and disease processes [183]. For instance, metagenomic analysis has revealed that specific microbial communities in the rumen are responsible for the breakdown of complex polysaccharides into simpler sugars, which are crucial for the cow’s energy supply. Genes encoding for glycoside hydrolases and polysaccharide lyases have been identified, illustrating the microbiome’s role in fiber digestion and energy metabolism [184].

Metatranscriptomic studies provide insights into how microbial gene expression varies with diet changes [185]. Pitta et al. [186] demonstrated that switching cattle from a forage-based diet to a grain-based diet significantly alters the expression of genes related to carbohydrate metabolism, highlighting the microbiome’s adaptive response to dietary shifts and its impact on host digestion and health.

Furthermore, metaproteomics has been used to characterize the protein profiles of the rumen microbiome, revealing enzymes involved in lignocellulose degradation, nitrogen fixation, and fatty acid metabolism [187,188].

Metabolomic analysis complements these approaches by identifying and quantifying the metabolites produced by the microbiome [189,190]. A study by Saleem et al. [191] utilized metabolomics to profile volatile fatty acids (VFAs) in the rumen, demonstrating the direct impact of microbial fermentation on the host’s energy balance and metabolic health. Likewise, the genital microbiome has been linked to semiochemical signaling between males and females in cattle [61,62].

## 9. Future Research Frontiers

### 9.1. Unexplored Dimensions of the Female Reproductive Microbiome

Future research in the realm of the female reproductive microbiome holds promise for uncovering unexplored dimensions that could revolutionize our understanding of reproductive health in cattle. While existing studies have shed light on the microbial composition of the female reproductive tract and its association with reproductive diseases, several aspects remain underexplored [23,44]. For instance, the functional roles of specific microbial species or communities in modulating reproductive processes, such as fertilization, implantation, and gestation, warrant further investigation [4,34,50]. Additionally, the impact of environmental factors, management practices, and host genetics on the female reproductive microbiome remains poorly understood [26]. By elucidating these interactions, it could be possible to identify potential interventions to optimize reproductive outcomes and enhance herd fertility.

Furthermore, advancements in high-throughput sequencing technologies, coupled with multi-omics approaches, offer exciting opportunities to delve deeper into the complexities of the reproductive microbiome. Integrating metagenomics, metatranscriptomics, metaproteomics, and metabolomics data can elucidate the functional dynamics of microbial communities and their interactions with the host environment [192].

### 9.2. Future Directions in Microbiome Manipulation

Future research in microbiome manipulation could explore novel techniques for targeted modulation of microbial communities to promote host health and productivity. This may involve the development of precision probiotics tailored to specific host–microbiome interactions, as well as the use of microbial consortia to achieve synergistic effects. Additionally, advancements in microbial engineering and synthetic biology could enable the design of custom microbial strains with enhanced probiotic properties or specific functional capabilities. Furthermore, investigating the impact of environmental factors, such as diet, housing conditions, and management practices, on the microbiome composition and function could provide valuable insights for optimizing microbiome manipulation strategies in livestock production.

### 9.3. Clinical and Commercial Applications

Exploring clinical and commercial applications of microbiome manipulation holds significant promise for various industries, including agriculture, healthcare, and biotechnology. In agriculture, targeted manipulation of the microbiome in livestock would improve animal health, enhance nutrient utilization, and mitigate the environmental impact of farming practices [193]. This could lead to increased productivity, reduced use of antibiotics, and improved sustainability in food production systems [194,195]. In healthcare, microbiome-based therapies such as probiotics, prebiotics, and genital microbiota transplantation could show promise in treating a wide range of diseases, including gastrointestinal disorders, metabolic diseases, and genital conditions, as has been proven with gut microbiomes [196]. Additionally, the development of microbiome-based diagnostics could revolutionize disease detection and personalized medicine approaches [197]. From a biotechnology perspective, the commercialization of microbiome-related products and services, such as microbial inoculants for agriculture, pharmaceuticals, and personalized nutrition, presents lucrative opportunities for innovation and economic growth.

### 9.4. Enhancing Existing and Novel Commercial Applications

The findings from ongoing research into the reproductive microbiome have the potential to significantly enhance both existing and novel commercial applications. For instance, precision probiotics designed based on specific microbial profiles could be developed to optimize reproductive health in livestock, leading to improved fertility rates and reduced costs associated with reproductive management [33,160]. In agriculture, such targeted interventions could also enhance animal growth and overall productivity [198]. Additionally, advancements in microbiome research could drive the development of new products, such as microbiome-modulating supplements and treatments for reproductive disorders, offering new revenue streams and market opportunities for companies in the agricultural and veterinary sectors [199]. The integration of microbiome data into precision farming practices could also lead to more sustainable and efficient livestock production systems, aligning with broader goals of environmental stewardship and food security [200].

## 10. Conclusions

The comprehensive review of the female reproductive microbiome underscores its pivotal role in shaping reproductive health outcomes in cattle. Advanced microbiome analysis techniques have illuminated the intricate dynamics of microbial communities within the genital tract and their significant impact on fertility. The evidence highlights how dysbiosis can contribute to reproductive disorders and reduced fertility. This underscores the critical need for strategies aimed at maintaining microbial equilibrium. The review also emphasizes the potential of microbiome manipulation strategies, including the use of precision probiotics, to address reproductive issues and enhance fertility in livestock. By exploring novel interventions and understanding their mechanisms, researchers can develop targeted approaches to optimize reproductive health. Future research should focus on unexplored aspects of the female reproductive microbiome, including the functional roles of specific microbial species and the influence of environmental and management factors. Advancements in this field have the potential to revolutionize reproductive management practices, improve herd fertility, and contribute to more sustainable agricultural practices. By advancing our understanding of microbial interactions and their implications for reproductive health, this research lays the groundwork for innovative solutions that can enhance productivity and sustainability in the agricultural sector.

## Figures and Tables

**Table 1:** Microbial functions and mechanisms in cattle reproduction. It summarizes the key microbial functions and mechanisms in cattle reproduction, highlighting the complex interactions between the microbiome and the host’s reproductive health. It covers communication through odor signals, immune modulation, pathogen defense, and microbial involvement in reproductive health, providing a comprehensive overview for better understanding and potential interventions.

Microbial Function	Mechanism	Example Bacteria	Impact on Reproduction
Communication and signaling	Odor-based signals Production of volatile fatty acids	*Staphylococcus*	Creates sex-specific odors for communication
Pathogen aggregation	Binding pathogens	*Lactobacillus rhamnosus GG*	Prevents pathogen adherence, inhibits *Escherichia coli*
Immune modulation	Regulation of immune function	*Lactobacilli*, *Bifidobacteria*	Modulates immune response, enhances defense
Antimicrobial compounds	Production of qhort-chain fatty acids, hydrogen peroxide, bacteriocins	Various *Lactobacilli*	Inhibits pathogens growth, reduces inflammation
Inflammation regulation	Cytokine production interference	*Lactobacillus*	Reduces proinflammatory cytokines induced by *Escherichia coli*

**Table 2:** Influence of genital microbiota on fertility and reproductive success in cattle. It outlines the role of specific bacterial mechanisms and their impacts on fertility and reproductive outcomes in cattle. Key points include the influence of the genital tract microbiome on sperm function and fertilization, the immune response triggered by pathogens, and the protective role of *Lactobacillus* against oxidative stress. The table also highlights the reduced microbial diversity during pregnancy and the beneficial presence of *Lactobacillus* for successful pregnancy outcomes.

Mechanism	Example Bacteria	Impact on Reproduction
Normal sperm functions and fertilization	-	Microbiome in the genital tract influences sperm function and fertilization capabilities.
Inflammatory response	*Escherichia coli*	Immune response to sperm and pathogens, involving TLRs in endometrial glands; *Escherichia coli* LPS leads to pseudopregnancy, luteal cysts, and anestrus.
Pathogen proliferation	*Trueperella pyogenes*, *Bovine gammaherpesvirus 4 (BoHV-4)*	Disruption of reproductive hormone secretion and folliculogenesis; pathogens impair fertility by damaging endometrial epithelial cells.
Oxidative stress protection	*Lactobacillus*	Enhances oocyte competency and fertilization; Lactobacillus produces phenolics, protects oocytes from oxidative stress, and suppresses eicosanoid production.
Pregnancy and microbiome diversity	*Firmicutes*, *Bacteroidetes*	Low diversity and abundance of microbiomes during pregnancy reduce dysbiosis and abortion risk; beneficial presence of *Lactobacillus* for successful pregnancy.

**Table 3:** Impact of pathogenic microbiota on reproductive health and disorders in cattle. It categorizes common reproductive disorders in cattle, identifies the associated causal bacteria, and explains the mechanisms by which these bacteria influence reproductive health. The table emphasizes the impact of bacterial diversity on the severity of these disorders and the interactions between pathogenic bacteria and the immune system.

Disorder	Causal Bacteria	Mechanism	Impact on Reproduction
Retained fetal membranes (RFM)	*Escherichia coli*, *Staphylococcus aureus*	Persistence of placental tissue creates a favorable environment for pathogen growth.	Leads to subfertility and increases the risk of uterine diseases like metritis and endometritis.
Metritis	*Fusobacterium*, *Bacteroides*, *Escherichia coli*	Deep inflammation of endometrium and myometrium; dominated by pathogenic bacteria.	Less diverse microbiome, symptoms linked to virulence factors, and symbiotic relationship between *Escherichia coli*, *Trueperella pyogenes*, and *Fusobacterium necrophorum* facilitates colonization and immune evasion.
Endometritis	*Bacteroides*, *Fusobacterium*, *Trueperella*	Superficial inflammation of the endometrium; lower microbial diversity.	Reduced abundance of beneficial bacteria like *Lactobacillus*; interactions between pathogens and Damage-associated molecular patterns (DAMP) lead to inflammatory responses; higher levels of *Anaerococcus*, *Corynebacterium*, and *Staphylococcus* increase risk.

**Table 6:** Probiotic strains and their adherence in endometrium. It highlights the significance of biofilm formation and the potential impact on reproductive health and disease management in cattle.

Probiotic Strain	Adherence Level	Biofilm Formation Ability	References
*Lactobacillus. sakei*, *Lactobacillus reuteri*	Low adherence	Low	[168]
*Pediococcus acidilactici*	Moderate to strong	High	[169]
*Lactobacillus rhamnosus*			[168]

**Table 7:** Overview of methodologies used in microbiome studies of the female reproductive tract in livestock.

Technique	Description	Application in Reproductive Microbiome Studies	Advantages	Limitations
High-Throughput Sequencing (HTS)	Sequencing of 16S rRNA genes to identify microbial communities.	Characterizes microbial diversity, richness, and shifts during reproductive stages.	Provides comprehensive microbial diversity analysis.	Limited functional information; identifies only DNA.
Shotgun Metagenomics	Sequencing entire genomes to study functional capabilities of microbial communities.	Investigates functional genes in reproductive tract microbes, such as metabolic potential.	Provides detailed gene-level insights.	Expensive and requires complex bioinformatics analysis.
Transcriptomics	Sequencing RNA to analyze active genes in microbial populations.	Identifies actively expressed genes within reproductive microbial communities.	Offers insights into microbial activity and response.	RNA is less stable and difficult to preserve.
Proteomics	Identification and quantification of microbial proteins.	Analyzes proteins produced by microbiome that impact reproductive health.	Provides functional understanding of microbial roles.	Requires sophisticated equipment and expertise.
Metabolomics	Analysis of small molecules and metabolites produced by microbes.	Studies metabolites influencing host–microbe interactions in the reproductive tract.	Identifies metabolites relevant to fertility and health.	Challenging to interpret in the context of host–microbe interactions.
Culture-Dependent Techniques	Isolation and cultivation of microorganisms from samples using growth media.	Studies viable and culturable bacteria in the reproductive tract, like E. coli and Fusobacterium.	Allows for testing microbial physiology and virulence.	Limited to culturable species; many microbes are unculturable.
MALDI-TOF MS	Mass spectrometry technique for rapid bacterial identification.	Identifies bacterial species from cultured samples.	Quick and precise identification of cultured bacteria.	Limited to organisms that can be cultured.
Custom Culture Media	Specialized media designed to mimic the reproductive tract environment.	Enhances the cultivation of fastidious and anaerobic microbes present in reproductive samples.	Allows for cultivation of previously unculturable microbes.	Still limited to microbes that can grow in artificial media.
Bioinformatics Pipelines (QIIME 2, DADA2, Mothur)	Software tools for analyzing sequencing data.	Analyzes sequencing data to determine microbial diversity, taxonomy, and functional pathways.	Automates large-scale data processing.	Requires bioinformatics expertise.

## Data Availability

Not applicable.

## References

[1] Otero C., Saavedra L., Silva de Ruiz C., Wilde O., Holgado A.R., Nader-Macias M.E. (2000). Vaginal bacterial microflora modifications during the growth of healthy cows. Lett. Appl. Microbiol..

[2] Otero C., Silva de Ruiz C., Ibañez R., Wilde O.R., de Ruiz Holgado A.A.P., Nader-Macias M.E. (1999). Lactobacilli and Enterococci Isolated from the Bovine Vagina during the Estrous cycle. Anaerobe.

[3] Swartz J.D., Lachman M., Westveer K., O’Neill T., Geary T., Kott R.W., Berardinelli J.G., Hatfield P.G., Thomson J.M., Roberts A. (2014). Characterization of the Vaginal Microbiota of Ewes and Cows Reveals a Unique Microbiota with Low Levels of Lactobacilli and Near-Neutral pH. Front. Vet. Sci..

[4] Laguardia-Nascimento M., Branco K.M., Gasparini M.R., Giannattasio-Ferraz S., Leite L.R., Araujo F.M., Salim A.C., Nicoli J.R., de Oliveira G.C., Barbosa-Stancioli E.F. (2015). Vaginal Microbiome Characterization of Nellore Cattle Using Metagenomic Analysis. PLoS ONE.

[5] Nesengani L.T., Wang J., Yang Y., Yang L., Lu W. (2017). Unravelling vaginal microbial genetic diversity and abundance between Holstein and Fleckvieh cattle. RSC Adv..

[6] Giannattasio-Ferraz S., Laguardia-Nascimento M., Gasparini M.R., Leite L.R., Araujo F.M.G., de Matos Salim A.C., de Oliveira A.P., Nicoli J.R., de Oliveira G.C., da Fonseca F.G. (2019). A common vaginal microbiota composition among breeds of Bos taurus indicus (Gyr and Nellore). Braz. J. Microbiol..

[7] Chen S.Y., Deng F., Zhang M., Jia X., Lai S.J. (2020). Characterization of Vaginal Microbiota Associated with Pregnancy Outcomes of Artificial Insemination in Dairy Cows. J. Microbiol. Biotechnol..

[8] Moreno C.G., Luque A.T., Galvão K.N., Otero M.C. (2022). Bacterial communities from vagina of dairy healthy heifers and cows with impaired reproductive performance. Res. Vet. Sci..

[9] Quadros D.L., Zanella R., Bondan C., Zanella G.C., Facioli F.L., da Silva A.N., Zanella E.L. (2020). Study of vaginal microbiota of Holstein cows submitted to an estrus synchronization protocol with the use of intravaginal progesterone device. Res. Vet. Sci..

[10] Quereda J.J., Barba M., Moce M.L., Gomis J., Jimenez-Trigos E., Garcia-Munoz A., Gomez-Martin A., Gonzalez-Torres P., Carbonetto B., Garcia-Rosello E. (2020). Vaginal Microbiota Changes during Estrous Cycle in Dairy Heifers. Front. Vet. Sci..

[11] Saini P., Singh M., Kumar P. (2019). Fungal endometritis in bovines. Open Vet. J..

[12] Appiah M.O., Wang J., Lu W. (2020). Microflora in the Reproductive Tract of Cattle: A Review. Agriculture.

[13] Sheldon I.M., Dobson H. (2004). Postpartum uterine health in cattle. Anim. Reprod. Sci..

[14] Singer E., Bushnell B., Coleman-Derr D., Bowman B., Bowers R.M., Levy A., Gies E.A., Cheng J.F., Copeland A., Klenk H.P. (2016). High-resolution phylogenetic microbial community profiling. ISME J..

[15] Wang Y., Wang J., Li H., Fu K., Pang B., Yang Y., Liu Y., Tian W., Cao R. (2018). Characterization of the cervical bacterial community in dairy cows with metritis and during different physiological phases. Theriogenology.

[16] Galvão K.N. (2012). Postpartum uterine diseases in dairy cows. Anim. Reprod. (AR).

[17] Deguillaume L., Geffre A., Desquilbet L., Dizien A., Thoumire S., Vorniere C., Constant F., Fournier R., Chastant-Maillard S. (2012). Effect of endocervical inflammation on days to conception in dairy cows. J. Dairy Sci..

[18] Adnane M., Meade K.G., O’Farrelly C. (2018). Cervico-vaginal mucus (CVM)—An accessible source of immunologically informative biomolecules. Vet. Res. Commun..

[19] Sheldon I.M., Cronin J.G., Bromfield J.J. (2019). Tolerance and innate immunity shape the development of postpartum uterine disease and the impact of endometritis in dairy cattle. Annu. Rev. Anim. Biosci..

[20] Karstrup C.C., Klitgaard K., Jensen T.K., Agerholm J.S., Pedersen H.G. (2017). Presence of bacteria in the endometrium and placentomes of pregnant cows. Theriogenology.

[21] Otero M.C., Morelli L., Nader-Macias M.E. (2006). Probiotic properties of vaginal lactic acid bacteria to prevent metritis in cattle. Lett. Appl. Microbiol..

[22] Ault T.B., Clemmons B.A., Reese S.T., Dantas F.G., Franco G.A., Smith T.P.L., Edwards J.L., Myer P.R., Pohler K.G. (2019). Uterine and vaginal bacterial community diversity prior to artificial insemination between pregnant and nonpregnant postpartum cows1. J. Anim. Sci..

[23] Rodrigues N.F., Kastle J., Coutinho T.J., Amorim A.T., Campos G.B., Santos V.M., Marques L.M., Timenetsky J., de Farias S.T. (2015). Qualitative analysis of the vaginal microbiota of healthy cattle and cattle with genital-tract disease. Genet. Mol. Res. GMR.

[24] Deng F., McClure M., Rorie R., Wang X., Chai J., Wei X., Lai S., Zhao J. (2019). The vaginal and fecal microbiomes are related to pregnancy status in beef heifers. J. Anim. Sci. Biotechnol..

[25] Messman R.D., Contreras-Correa Z.E., Paz H.A., Perry G., Lemley C.O. (2020). Vaginal bacterial community composition and concentrations of estradiol at the time of artificial insemination in Brangus heifers. J. Anim. Sci..

[26] Adnane M., Chapwanya A. (2022). A Review of the Diversity of the Genital Tract Microbiome and Implications for Fertility of Cattle. Animals.

[27] Moore S.G., Ericsson A.C., Poock S.E., Melendez P., Lucy M.C. (2017). Hot topic: 16S rRNA gene sequencing reveals the microbiome of the virgin and pregnant bovine uterus. J. Dairy Sci..

[28] Jeon S.J., Cunha F., Vieira-Neto A., Bicalho R.C., Lima S., Bicalho M.L., Galvao K.N. (2017). Blood as a route of transmission of uterine pathogens from the gut to the uterus in cows. Microbiome.

[29] van der Burgt G., Clark W., Knight R., Colles K. (2007). Cattle fertility problems and Histophilus somni. Vet. Rec..

[30] Nicholas R., Ayling R., McAuliffe L. (2008). Mycoplasma Diseases of Ruminants.

[31] Adegboye D.S., Halbur P.G., Nutsch R.G., Kadlec R.G., Rosenbusch R.F. (1996). Mycoplasma bovis-associated pneumonia and arthritis complicated with pyogranulomatous tenosynovitis in calves. J. Am. Vet. Med. Assoc..

[32] Bicalho M.L., Machado V.S., Oikonomou G., Gilbert R.O., Bicalho R.C. (2012). Association between virulence factors of Escherichia coli, Fusobacterium necrophorum, and Arcanobacterium pyogenes and uterine diseases of dairy cows. Vet. Microbiol..

[33] Luecke S.M., Webb E.M., Dahlen C.R., Reynolds L.P., Amat S. (2022). Seminal and vagino-uterine microbiome and their individual and interactive effects on cattle fertility. Front. Microbiol..

[34] Carneiro L.C., Cronin J.G., Sheldon I.M. (2016). Mechanisms linking bacterial infections of the bovine endometrium to disease and infertility. Reprod. Biol..

[35] Galvao K.N., Bicalho R.C., Jeon S.J. (2019). Symposium review: The uterine microbiome associated with the development of uterine disease in dairy cows. J. Dairy Sci..

[36] Hashem N.M., Gonzalez-Bulnes A. (2022). The Use of Probiotics for Management and Improvement of Reproductive Eubiosis and Function. Nutrients.

[37] Esposito G., Raffrenato E., Lukamba S.D., Adnane M., Irons P.C., Cormican P., Tasara T., Chapwanya A. (2020). Characterization of metabolic and inflammatory profiles of transition dairy cows fed an energy-restricted diet. J. Anim. Sci..

[38] El-Jakee J., Ahmed W., El-Seedy F., El-Moez S. (2008). Bacterial profile of the genital tract in female-buffalo during the different reproductive stages. Glob. Vet..

[39] Wang J., Liu C., Nesengani L.T., Gong Y., Yang Y., Yang L., Lu W. (2019). Comparison of vaginal microbial community structure of beef cattle between luteal phase and follicular phase. Indian J. Anim. Res..

[40] Petit T., Spergser J., Rosengarten R., Aurich J. (2009). Prevalence of potentially pathogenic bacteria as genital pathogens in dairy cattle. Reprod. Domest. Anim. Zuchthyg..

[41] Udhayavel S., Malmarugan S., Palanisamy K., Rajeswar J. (2013). Antibiogram pattern of bacteria causing endometritis in cows. Vet. World.

[42] Onnureddy K., Vengalrao Y., Mohanty T.K., Singh D. (2013). Metagenomic Analysis of Uterine Microbiota in Postpartum Normal and Endometritic Water Buffaloes (*Bubalus bubalis*). J. Buffalo Sci..

[43] Pascottini O.B., Van Schyndel S.J., Spricigo J.F.W., Rousseau J., Weese J.S., LeBlanc S.J. (2020). Dynamics of uterine microbiota in postpartum dairy cows with clinical or subclinical endometritis. Sci. Rep..

[44] Miranda-CasoLuengo R., Lu J., Williams E.J., Miranda-CasoLuengo A.A., Carrington S.D., Evans A.C.O., Meijer W.G. (2019). Delayed differentiation of vaginal and uterine microbiomes in dairy cows developing postpartum endometritis. PLoS ONE.

[45] Moore S.G., Ericsson A.C., Behura S.K., Lamberson W.R., Evans T.J., McCabe M.S., Poock S.E., Lucy M.C. (2019). Concurrent and long-term associations between the endometrial microbiota and endometrial transcriptome in postpartum dairy cows. BMC Genom..

[46] Perry G.A., Perry B.L. (2008). Effect of preovulatory concentrations of estradiol and initiation of standing estrus on uterine pH in beef cows. Domest. Anim. Endocrinol..

[47] Owens C.E., Daniels K.M., Ealy A.D., Knowlton K.F., Cockrum R.R. (2020). Graduate Student Literature Review: Potential mechanisms of interaction between bacteria and the reproductive tract of dairy cattle. J. Dairy Sci..

[48] Sheldon I.M., Price S.B., Cronin J., Gilbert R.O., Gadsby J.E. (2009). Mechanisms of infertility associated with clinical and subclinical endometritis in high producing dairy cattle. Reprod. Domest. Anim. Zuchthyg..

[49] Sheldon I.M., Noakes D.E., Rycroft A.N., Pfeiffer D.U., Dobson H. (2002). Influence of uterine bacterial contamination after parturition on ovarian dominant follicle selection and follicle growth and function in cattle. Reproduction.

[50] Herath S., Lilly S.T., Fischer D.P., Williams E.J., Dobson H., Bryant C.E., Sheldon I.M. (2009). Bacterial lipopolysaccharide induces an endocrine switch from prostaglandin F2alpha to prostaglandin E2 in bovine endometrium. Endocrinology.

[51] Cronin J.G., Turner M.L., Goetze L., Bryant C.E., Sheldon I.M. (2012). Toll-like receptor 4 and MYD88-dependent signaling mechanisms of the innate immune system are essential for the response to lipopolysaccharide by epithelial and stromal cells of the bovine endometrium. Biol. Reprod..

[52] Herath S., Williams E.J., Lilly S.T., Gilbert R.O., Dobson H., Bryant C.E., Sheldon I.M. (2007). Ovarian follicular cells have innate immune capabilities that modulate their endocrine function. Reproduction.

[53] Bromfield J.J., Santos J.E., Block J., Williams R.S., Sheldon I.M. (2015). Physiology and Endocrinology Symposium: Uterine infection: Linking infection and innate immunity with infertility in the high-producing dairy cow. J. Anim. Sci..

[54] Williams E.J., Fischer D.P., Noakes D.E., England G.C., Rycroft A., Dobson H., Sheldon I.M. (2007). The relationship between uterine pathogen growth density and ovarian function in the postpartum dairy cow. Theriogenology.

[55] Bonnett B.N., Martin S.W., Gannon V.P., Miller R.B., Etherington W.G. (1991). Endometrial biopsy in Holstein-Friesian dairy cows. III. Bacteriological analysis and correlations with histological findings. Can. J. Vet. Res..

[56] Sakai M., Ishiyama A., Tabata M., Sasaki Y., Yoneda S., Shiozaki A., Saito S. (2004). Relationship between cervical mucus interleukin-8 concentrations and vaginal bacteria in pregnancy. Am. J. Reprod. Immunol..

[57] Jadon R.S., Dhaliwal G.S., Jand S.K. (2005). Prevalence of aerobic and anaerobic uterine bacteria during peripartum period in normal and dystocia-affected buffaloes. Anim. Reprod. Sci..

[58] Raza S., Rabbani M., Ahmad N., Sheikh A.A., Muhammad K., Akhtar F., Rehman H.U. (2013). Uterine microbial flora of Nili-Ravi buffalo during estrus and its relationship with pregnancy rate in Pakistan. Editor. Board.

[59] Kumar M., Choudhary S. (2019). Age estimation using pulp tooth area ratio in North Indian population. J. Indian Acad. Oral Med. Radiol..

[60] Aagaard K., Riehle K., Ma J., Segata N., Mistretta T.A., Coarfa C., Raza S., Rosenbaum S., Van den Veyver I., Milosavljevic A. (2012). A metagenomic approach to characterization of the vaginal microbiome signature in pregnancy. PLoS ONE.

[61] Archunan G., Rajanarayanan S., Karthikeyan K., Mucignat-Caretta C. (2014). Cattle Pheromones. Neurobiology of Chemical Communication.

[62] Srinivasan M., Adnane M., Archunan G. (2021). Significance of cervico-vaginal microbes in bovine reproduction and pheromone production—A hypothetical review. Res. Vet. Sci..

[63] Buck L.B. (2000). The molecular architecture of odor and pheromone sensing in mammals. Cell.

[64] Root Kustritz M.V. (2005). Reproductive behavior of small animals. Theriogenology.

[65] Mertens P.A. (2006). Reproductive and sexual behavioral problems in dogs. Theriogenology.

[66] Ziegler T.E., Epple G., Snowdon C.T., Porter T.A., Belcher A.M., Küderling I. (1993). Detection of the chemical signals of ovulation in the cotton-top tamarin, Saguinus oedipus. Anim. Behav..

[67] Ezenwa V.O., Williams A.E. (2014). Microbes and animal olfactory communication: Where do we go from here?. Bioessays.

[68] Merkx J., Slob A.K., van der Werff ten Bosch J.J. (1988). Vaginal bacterial flora partially determines sexual attractivity of female rats. Physiol. Behav..

[69] Wong A.C., Holmes A., Ponton F., Lihoreau M., Wilson K., Raubenheimer D., Simpson S.J. (2015). Behavioral Microbiomics: A Multi-Dimensional Approach to Microbial Influence on Behavior. Front. Microbiol..

[70] Natsch A., Buettner A. (2017). Biochemistry and Genetics of Human Axilla Odor. Springer Handbook of Odor.

[71] Leclaire S., Jacob S., Greene L.K., Dubay G.R., Drea C.M. (2017). Social odours covary with bacterial community in the anal secretions of wild meerkats. Sci. Rep..

[72] Theis K.R., Venkataraman A., Dycus J.A., Koonter K.D., Schmitt-Matzen E.N., Wagner A.P., Holekamp K.E., Schmidt T.M. (2013). Symbiotic bacteria appear to mediate hyena social odors. Proc. Natl. Acad. Sci. USA.

[73] Theis K.R., Schmidt T.M., Holekamp K.E. (2012). Evidence for a bacterial mechanism for group-specific social odors among hyenas. Sci. Rep..

[74] Sengupta R., Altermann E., Anderson R.C., McNabb W.C., Moughan P.J., Roy N.C. (2013). The role of cell surface architecture of lactobacilli in host-microbe interactions in the gastrointestinal tract. Mediat. Inflamm..

[75] Bermudez-Brito M., Plaza-Diaz J., Munoz-Quezada S., Gomez-Llorente C., Gil A. (2012). Probiotic mechanisms of action. Ann. Nutr. Metab..

[76] Ametaj B.N., Iqbal S., Selami F., Odhiambo J.F., Wang Y., Ganzle M.G., Dunn S.M., Zebeli Q. (2014). Intravaginal administration of lactic acid bacteria modulated the incidence of purulent vaginal discharges, plasma haptoglobin concentrations, and milk production in dairy cows. Res. Vet. Sci..

[77] Vinderola G., Ouwehand A.C., Salminen S., von Wright A. (2019). Lactic Acid Bacteria.

[78] Makras L., Triantafyllou V., Fayol-Messaoudi D., Adriany T., Zoumpopoulou G., Tsakalidou E., Servin A., De Vuyst L. (2006). Kinetic analysis of the antibacterial activity of probiotic lactobacilli towards *Salmonella enterica* serovar Typhimurium reveals a role for lactic acid and other inhibitory compounds. Res. Microbiol..

[79] Greene J.D., Klaenhammer T.R. (1994). Factors involved in adherence of lactobacilli to human Caco-2 cells. Appl. Environ. Microbiol..

[80] Hassan M., Kjos M., Nes I.F., Diep D.B., Lotfipour F. (2012). Natural antimicrobial peptides from bacteria: Characteristics and potential applications to fight against antibiotic resistance. J. Appl. Microbiol..

[81] Yildirim Z., Winters D.K., Johnson M.G. (1999). Purification, amino acid sequence and mode of action of bifidocin B produced by Bifidobacterium bifidum NCFB 1454. J. Appl. Microbiol..

[82] Furrie E., Macfarlane S., Kennedy A., Cummings J.H., Walsh S.V., O’Neil D.A., Macfarlane G.T. (2005). Synbiotic therapy (Bifidobacterium longum/Synergy 1) initiates resolution of inflammation in patients with active ulcerative colitis: A randomised controlled pilot trial. Gut.

[83] Prema P., Smila D., Palavesam A., Immanuel G. (2008). Production and Characterization of an Antifungal Compound (3-Phenyllactic Acid) Produced by Lactobacillus plantarum Strain. Food Bioprocess Technol..

[84] Niku-Paavola M.L., Laitila A., Mattila-Sandholm T., Haikara A. (1999). New types of antimicrobial compounds produced by Lactobacillus plantarum. J. Appl. Microbiol..

[85] Averesch N.J.H., Kromer J.O. (2018). Metabolic Engineering of the Shikimate Pathway for Production of Aromatics and Derived Compounds-Present and Future Strain Construction Strategies. Front. Bioeng. Biotechnol..

[86] Buhaescu I., Izzedine H. (2007). Mevalonate pathway: A review of clinical and therapeutical implications. Clin. Biochem..

[87] Dal Bello F., Clarke C.I., Ryan L.A.M., Ulmer H., Schober T.J., Ström K., Sjögren J., van Sinderen D., Schnürer J., Arendt E.K. (2007). Improvement of the quality and shelf life of wheat bread by fermentation with the antifungal strain Lactobacillus plantarum FST 1.7. J. Cereal Sci..

[88] Liu M., Wu Q., Wang M., Fu Y., Wang J. (2016). Lactobacillus rhamnosus GR-1 Limits Escherichia coli-Induced Inflammatory Responses via Attenuating MyD88-Dependent and MyD88-Independent Pathway Activation in Bovine Endometrial Epithelial Cells. Inflammation.

[89] Genis S., Sanchez-Chardi A., Bach A., Fabregas F., Aris A. (2017). A combination of lactic acid bacteria regulates Escherichia coli infection and inflammation of the bovine endometrium. J. Dairy Sci..

[90] Otero M.C., Nader-Macias M.E. (2006). Inhibition of Staphylococcus aureus by H_2_O_2_-producing Lactobacillus gasseri isolated from the vaginal tract of cattle. Anim. Reprod. Sci..

[91] Alakomi H.L., Skytta E., Saarela M., Mattila-Sandholm T., Latva-Kala K., Helander I.M. (2000). Lactic acid permeabilizes gram-negative bacteria by disrupting the outer membrane. Appl. Environ. Microbiol..

[92] Dominguez-Bello M.G., Costello E.K., Contreras M., Magris M., Hidalgo G., Fierer N., Knight R. (2010). Delivery mode shapes the acquisition and structure of the initial microbiota across multiple body habitats in newborns. Proc. Natl. Acad. Sci. USA.

[93] Sheldon I.M., Cronin J., Goetze L., Donofrio G., Schuberth H.J. (2009). Defining postpartum uterine disease and the mechanisms of infection and immunity in the female reproductive tract in cattle. Biol. Reprod..

[94] LeBlanc S.J., Duffield T.F., Leslie K.E., Bateman K.G., Keefe G.P., Walton J.S., Johnson W.H. (2002). Defining and diagnosing postpartum clinical endometritis and its impact on reproductive performance in dairy cows. J. Dairy Sci..

[95] Peng Y., Wang Y., Hang S., Zhu W. (2013). Microbial diversity in uterus of healthy and metritic postpartum Holstein dairy cows. Folia Microbiol..

[96] Elkjaer K., Ancker M.L., Gustafsson H., Friggens N.C., Waldmann A., Molbak L., Callesen H. (2013). Uterine bacterial flora in postpartum Danish Holstein dairy cows determined using DNA-based fingerprinting: Correlation to uterine condition and calving management. Anim. Reprod. Sci..

[97] Bicalho M.L.S., Santin T., Rodrigues M.X., Marques C.E., Lima S.F., Bicalho R.C. (2017). Dynamics of the microbiota found in the vaginas of dairy cows during the transition period: Associations with uterine diseases and reproductive outcome. J. Dairy Sci..

[98] Akthar I., Suarez S.S., Morillo V.A., Sasaki M., Ezz M.A., Takahashi K.I., Shimada M., Marey M.A., Miyamoto A. (2020). Sperm enter glands of preovulatory bovine endometrial explants and initiate inflammation. Reproduction.

[99] Sicsic R., Goshen T., Dutta R., Kedem-Vaanunu N., Kaplan-Shabtai V., Pasternak Z., Gottlieb Y., Shpigel N.Y., Raz T. (2018). Microbial communities and inflammatory response in the endometrium differ between normal and metritic dairy cows at 5–10 days post-partum. Vet. Res..

[100] Thulasiraman S., Gunasekar M., Narayansamy A., Sampathkumar K.U., Kumar R., Alam K. (2024). Uterine Infections/Metritis. Periparturient Diseases of Cattle.

[101] Vallejo-Timaran D.A., Reyes J., Gilbert R.O., Lefebvre R.C., Palacio-Baena L.G., Maldonado-Estrada J.G. (2021). Incidence, clinical patterns, and risk factors of postpartum uterine diseases in dairy cows from high-altitude tropical herds. J. Dairy Sci..

[102] Donofrio G., Herath S., Sartori C., Cavirani S., Flammini C.F., Sheldon I.M. (2007). Bovine herpesvirus 4 is tropic for bovine endometrial cells and modulates endocrine function. Reproduction.

[103] Davies D., Meade K.G., Herath S., Eckersall P.D., Gonzalez D., White J.O., Conlan R.S., O’Farrelly C., Sheldon I.M. (2008). Toll-like receptor and antimicrobial peptide expression in the bovine endometrium. Reprod. Biol. Endocrinol..

[104] Kassé F., Fairbrother J., Dubuc J. (2016). Relationship between Escherichia coli virulence factors and postpartum metritis in dairy cows. J. Dairy Sci..

[105] Schust D., Anderson D., Hill J. (1996). Progesterone-induced immunosuppression is not mediated through the progesterone receptor. Hum. Reprod..

[106] Watson E.D., Stokes C.R., David J.S., Bourne F.J. (1987). Effect of ovarian hormones on promotion of bactericidal activity by uterine secretions of ovariectomized mares. J. Reprod. Fertil..

[107] Ault T.B., Clemmons B.A., Reese S.T., Dantas F.G., Franco G.A., Smith T.P., Edwards J.L., Myer P.R., Pohler K.G. (2019). Bacterial taxonomic composition of the postpartum cow uterus and vagina prior to artificial insemination. J. Anim. Sci..

[108] Guerreiro T.M., Goncalves R.F., Melo C., de Oliveira D.N., Lima E.O., Visintin J.A., de Achilles M.A., Catharino R.R. (2018). A Metabolomic Overview of Follicular Fluid in Cows. Front. Vet. Sci..

[109] Martins N., Barros L., Ferreira I.C.F.R. (2016). In vivo antioxidant activity of phenolic compounds: Facts and gaps. Trends Food Sci. Technol..

[110] Rodriguez H., Curiel J.A., Landete J.M., de las Rivas B., Lopez de Felipe F., Gomez-Cordoves C., Mancheno J.M., Munoz R. (2009). Food phenolics and lactic acid bacteria. Int. J. Food Microbiol..

[111] Jabbour H.N., Sales K.J., Catalano R.D., Norman J.E. (2009). Inflammatory pathways in female reproductive health and disease. Reproduction.

[112] Li C.-X., Jiang X.-C., Qiu Y.-J., Xu J.-H. (2015). Identification of a new thermostable and alkali-tolerant α-carbonic anhydrase from *Lactobacillus delbrueckii* as a biocatalyst for CO_2_ biomineralization. Bioresour. Bioprocess..

[113] Visconti P.E., Krapf D., de la Vega-Beltran J.L., Acevedo J.J., Darszon A. (2011). Ion channels, phosphorylation and mammalian sperm capacitation. Asian J. Androl..

[114] Fulop V., Demeter J., Cseh A. (2021). Significance and effects of prenatal and postnatal microbiome in the period of early individual development and options for interventional treatment. Orvosi Hetil..

[115] Moreno I., Codoner F.M., Vilella F., Valbuena D., Martinez-Blanch J.F., Jimenez-Almazan J., Alonso R., Alama P., Remohi J., Pellicer A. (2016). Evidence that the endometrial microbiota has an effect on implantation success or failure. Am. J. Obs. Gynecol..

[116] Vacca P., Cantoni C., Vitale M., Prato C., Canegallo F., Fenoglio D., Ragni N., Moretta L., Mingari M.C. (2010). Crosstalk between decidual NK and CD14+ myelomonocytic cells results in induction of Tregs and immunosuppression. Proc. Natl. Acad. Sci. USA.

[117] Clemmons B.A., Reese S.T., Dantas F.G., Franco G.A., Smith T.P.L., Adeyosoye O.I., Pohler K.G., Myer P.R. (2017). Vaginal and Uterine Bacterial Communities in Postpartum Lactating Cows. Front. Microbiol..

[118] Sheldon I.M., Williams E.J., Miller A.N., Nash D.M., Herath S. (2008). Uterine diseases in cattle after parturition. Vet. J..

[119] Ong C.T., Turni C., Blackall P.J., Boe-Hansen G., Hayes B.J., Tabor A.E. (2021). Interrogating the bovine reproductive tract metagenomes using culture-independent approaches: A systematic review. Anim. Microbiome.

[120] Wang M.L., Liu M.C., Xu J., An L.G., Wang J.F., Zhu Y.H. (2018). Uterine Microbiota of Dairy Cows With Clinical and Subclinical Endometritis. Front. Microbiol..

[121] Potter T.J., Guitian J., Fishwick J., Gordon P.J., Sheldon I.M. (2010). Risk factors for clinical endometritis in postpartum dairy cattle. Theriogenology.

[122] Al-Zubaidi S.F.A., Hasson S.O., Ajeel H.H. (2017). Isolation and identification of the bacteria associated with the retained fetal membranes in cows by PCR technique in Babylon-Iraq. Euphrates J. Agric. Sci..

[123] Kim I.H., Kang H.G. (2003). Risk factors for postpartum endometritis and the effect of endometritis on reproductive performance in dairy cows in Korea. J. Reprod. Dev..

[124] Adnane M., Kaidi R., Hanzen C., England G.C.W. (2017). Risk factors of clinical and subclinical endometritis in cattle: A review. Turk. J. Vet. Anim. Sci..

[125] Sheldon I.M., Lewis G.S., LeBlanc S., Gilbert R.O. (2006). Defining postpartum uterine disease in cattle. Theriogenology.

[126] Yamamura F., Sugiura T., Munby M., Shiokura Y., Murata R., Nakamura T., Fujiki J., Iwano H. (2022). Relationship between Escherichia coli virulence factors, notably kpsMTII, and symptoms of clinical metritis and endometritis in dairy cows. J. Vet. Med. Sci..

[127] Ungerfeld R., Silva L. (2005). The presence of normal vaginal flora is necessary for normal sexual attractiveness of estrous ewes. Appl. Anim. Behav. Sci..

[128] Jemiolo B., Miller K.V., Wiesler D., Jelinek I., Novotny M., Marchinton R.L. (1995). Putative chemical signals from white-tailed deer (*Odocoileus virginianus*). Urinary and vaginal mucus volatiles excreted by females during breeding season. J. Chem. Ecol..

[129] Bonsall R.W., Michael R.P. (1971). Volatile constituents of primate vaginal secretions. J. Reprod. Fertil..

[130] Michael R.P., Bonsall R.W., Warner P., Denton D.A., Coghlan J.P. (1975). Primate Sexual Pheromones. Olfaction and Taste: 5th Symposium.

[131] Clarke P.M., Barrett L., Henzi S.P. (2009). What role do olfactory cues play in chacma baboon mating?. Am. J. Primatol..

[132] Miller E.A., Livermore J.A., Alberts S.C., Tung J., Archie E.A. (2017). Ovarian cycling and reproductive state shape the vaginal microbiota in wild baboons. Microbiome.

[133] Valle G.R., Toledo Junior J.C., de Figueiredo C.B. (2006). Detection of Simonsiella species in the vagina of a bitch in heat. Vet. Rec..

[134] Koziol J.H., Sheets T., Wickware C.L., Johnson T.A. (2022). Composition and diversity of the seminal microbiota in bulls and its association with semen parameters. Theriogenology.

[135] Givens M.D., Marley M.S. (2008). Pathogens that cause infertility of bulls or transmission via semen. Theriogenology.

[136] Poole R.K., Soffa D.R., McAnally B.E., Smith M.S., Hickman-Brown K.J., Stockland E.L. (2023). Reproductive Microbiomes in Domestic Livestock: Insights Utilizing 16S rRNA Gene Amplicon Community Sequencing. Animals.

[137] Sannat C., Nair A., Sahu S.B., Sahasrabudhe S.A., Kumar A., Gupta A.K., Shende R.K. (2015). Effect of species, breed, and age on bacterial load in bovine and bubaline semen. Vet. World.

[138] González-Marín C., Roy R., López-Fernández C., Diez B., Carabaño M., Fernández J.L., Kjelland M., Moreno J.F., Gosálvez J. (2011). Bacteria in bovine semen can increase sperm DNA fragmentation rates: A kinetic experimental approach. Anim. Reprod. Sci..

[139] Medo J., Ziarovska J., Duracka M., Tvrda E., Banas S., Gabor M., Kysel M., Kacaniova M. (2021). Core Microbiome of Slovak Holstein Friesian Breeding Bulls’ Semen. Animals.

[140] del Porto G.B., Derrick F.C., Bannister E.R. (1975). Bacterial effect on sperm motility. Urology.

[141] Cagnoli C.I., Chiapparrone M.L., Cacciato C.S., Rodriguez M.G., Aller J.F., Catena M.D.C. (2020). Effects of Campylobacter fetus on bull sperm quality. Microb. Pathog..

[142] Marchiani S., Baccani I., Tamburrino L., Mattiuz G., Nicolo S., Bonaiuto C., Panico C., Vignozzi L., Antonelli A., Rossolini G.M. (2021). Effects of common Gram-negative pathogens causing male genitourinary-tract infections on human sperm functions. Sci. Rep..

[143] Eini F., Kutenaei M.A., Zareei F., Dastjerdi Z.S., Shirzeyli M.H., Salehi E. (2021). Effect of bacterial infection on sperm quality and DNA fragmentation in subfertile men with Leukocytospermia. BMC Mol. Cell Biol..

[144] Maretti C., Cavallini G. (2017). The association of a probiotic with a prebiotic (Flortec, Bracco) to improve the quality/quantity of spermatozoa in infertile patients with idiopathic oligoasthenoteratospermia: A pilot study. Andrology.

[145] Valcarce D.G., Genoves S., Riesco M.F., Martorell P., Herraez M.P., Ramon D., Robles V. (2017). Probiotic administration improves sperm quality in asthenozoospermic human donors. Benef. Microbes.

[146] Farahani L., Tharakan T., Yap T., Ramsay J.W., Jayasena C.N., Minhas S. (2021). The semen microbiome and its impact on sperm function and male fertility: A systematic review and meta-analysis. Andrology.

[147] Parker A.M., House J.K., Hazelton M.S., Bosward K.L., Sheehy P.A. (2017). Comparison of culture and a multiplex probe PCR for identifying Mycoplasma species in bovine milk, semen and swab samples. PLoS ONE.

[148] Rana P., Singh M., Dhaka Y. (2020). In vitro antibiogram of bacterial isolates from preputial washings and cow bull semen. Haryana Vet..

[149] LeBlanc S.J. (2008). Postpartum uterine disease and dairy herd reproductive performance: A review. Vet. J..

[150] El-Khadrawy H., Ahmed W.M., Zaabal M., Hanafi E.M. (2015). Strategies for diagnosis and treatment of uterine infection in bovines. Glob. Vet..

[151] De Seta F., Parazzini F., De Leo R., Banco R., Maso G.P., De Santo D., Sartore A., Stabile G., Inglese S., Tonon M. (2014). Lactobacillus plantarum P17630 for preventing Candida vaginitis recurrence: A retrospective comparative study. Eur. J. Obs. Gynecol. Reprod. Biol..

[152] Stapleton A.E., Au-Yeung M., Hooton T.M., Fredricks D.N., Roberts P.L., Czaja C.A., Yarova-Yarovaya Y., Fiedler T., Cox M., Stamm W.E. (2011). Randomized, placebo-controlled phase 2 trial of a Lactobacillus crispatus probiotic given intravaginally for prevention of recurrent urinary tract infection. Clin. Infect. Dis..

[153] Genis S., Cerri R.L.A., Bach A., Silper B.F., Baylao M., Denis-Robichaud J., Aris A. (2018). Pre-calving Intravaginal Administration of Lactic Acid Bacteria Reduces Metritis Prevalence and Regulates Blood Neutrophil Gene Expression after Calving in Dairy Cattle. Front. Vet. Sci..

[154] Abdul-Abbas S.J., Al-Badran A.E., Al-bayyar A.H.A., Al-Sherifi H.R. (2016). Isolation and identification of Alocalstrain of probiotic bacterial Actobacillus plantarum and studied the tolerance ability for different levels of pH. Basrah J. Vet. Res..

[155] Gorzelak M.A., Gill S.K., Tasnim N., Ahmadi-Vand Z., Jay M., Gibson D.L. (2015). Methods for improving human gut microbiome data by reducing variability through sample processing and storage of stool. PLoS ONE.

[156] Deng Q., Odhiambo J.F., Farooq U., Lam T., Dunn S.M., Ametaj B.N. (2014). Intravaginal lactic Acid bacteria modulated local and systemic immune responses and lowered the incidence of uterine infections in periparturient dairy cows. PLoS ONE.

[157] Pellegrino M., Berardo N., Giraudo J., Nader-Macías M., Bogni C. (2017). Bovine mastitis prevention: Humoral and cellular response of dairy cows inoculated with lactic acid bacteria at the dry-off period. Benef. Microbes.

[158] Madureira A.M.L., Burnett T.A., Boyd C.T., Baylao M., Cerri R.L.A. (2023). Use of intravaginal lactic acid bacteria prepartum as an approach for preventing uterine disease and its association with fertility of lactating dairy cows. J. Dairy Sci..

[159] Abbas A.K., Lichtman A.H., Pillai S. (2007). Cellular and Molecular Immunology.

[160] Adnane M., Whiston R., Tasara T., Bleul U., Chapwanya A. (2024). Harnessing Vaginal Probiotics for Enhanced Management of Uterine Disease and Reproductive Performance in Dairy Cows: A Conceptual Review. Animals.

[161] Oliver S.P., Murinda S.E. (2012). Antimicrobial resistance of mastitis pathogens. Vet. Clin. N. Am. Food Anim. Pract..

[162] Mathys S., von Ah U., Lacroix C., Staub E., Mini R., Cereghetti T., Meile L. (2007). Detection of the pediocin gene pedA in strains from human faeces by real-time PCR and characterization of Pediococcus acidilactici UVA1. BMC Biotechnol..

[163] Wang Y., Ametaj B.N., Ambrose D.J., Ganzle M.G. (2013). Characterisation of the bacterial microbiota of the vagina of dairy cows and isolation of pediocin-producing Pediococcus acidilactici. BMC Microbiol..

[164] Peter S., Gartner M.A., Michel G., Ibrahim M., Klopfleisch R., Lubke-Becker A., Jung M., Einspanier R., Gabler C. (2018). Influence of intrauterine administration of Lactobacillus buchneri on reproductive performance and pro-inflammatory endometrial mRNA expression of cows with subclinical endometritis. Sci. Rep..

[165] Hossain M.E., Kim G.M., Lee S.K., Yang C.J. (2012). Growth performance, meat yield, oxidative stability, and Fatty Acid composition of meat from broilers fed diets supplemented with a medicinal plant and probiotics. Asian-Australas. J. Anim. Sci..

[166] Niu C., Cheng C., Liu Y., Huang S., Fu Y., Li P. (2019). Transcriptome Profiling Analysis of Bovine Vaginal Epithelial Cell Response to an Isolated Lactobacillus Strain. mSystems.

[167] Vieco-Saiz N., Belguesmia Y., Raspoet R., Auclair E., Gancel F., Kempf I., Drider D. (2019). Benefits and inputs from lactic acid bacteria and their bacteriocins as alternatives to antibiotic growth promoters during food-animal production. Front. Microbiol..

[168] Genis S., Bach A., Fabregas F., Aris A. (2016). Potential of lactic acid bacteria at regulating Escherichia coli infection and inflammation of bovine endometrium. Theriogenology.

[169] Atshan S.S., Shamsudin M.N., Lung L.T., Sekawi Z., Ghaznavi-Rad E., Pei C.P. (2012). Comparative characterisation of genotypically different clones of MRSA in the production of biofilms. J. Biomed. Biotechnol..

[170] Webb E.M., Holman D.B., Schmidt K.N., Pun B., Sedivec K.K., Hurlbert J.L., Bochantin K.A., Ward A.K., Dahlen C.R., Amat S. (2023). Sequencing and culture-based characterization of the vaginal and uterine microbiota in beef cattle that became pregnant or remained open following artificial insemination. Microbiol. Spectr..

[171] Kero K., Hieta N., Kallonen T., Ahtikoski A., Laine H.K., Rautava J., Munukka E. (2023). Optimal sampling and analysis methods for clinical diagnostics of vaginal microbiome. Eur. J. Clin. Microbiol. Infect. Dis..

[172] Lyu R., Qu Y., Divaris K., Wu D. (2024). Methodological Considerations in Longitudinal Analyses of Microbiome Data: A Comprehensive Review. Genes.

[173] de Medeiros Garcia Torres M., Lanza D.C.F. (2024). A Standard Pipeline for Analyzing the Endometrial Microbiome. Reprod. Sci..

[174] Koedooder R., Mackens S., Budding A., Fares D., Blockeel C., Laven J., Schoenmakers S. (2019). Identification and evaluation of the microbiome in the female and male reproductive tracts. Hum. Reprod. Update.

[175] Wagener K., Grunert T., Prunner I., Ehling-Schulz M., Drillich M. (2014). Dynamics of uterine infections with Escherichia coli, Streptococcus uberis and Trueperella pyogenes in post-partum dairy cows and their association with clinical endometritis. Vet. J..

[176] Osawa T. (2021). Predisposing factors, diagnostic and therapeutic aspects of persistent endometritis in postpartum cows. J. Reprod. Dev..

[177] Howe S., Liu Z., Zuo B., Zhao J. (2024). Culturomics: A critical approach in studying the roles of human and animal microbiota. Anim. Nutr..

[178] Kleiner M., Thorson E., Sharp C.E., Dong X., Liu D., Li C., Strous M. (2017). Assessing species biomass contributions in microbial communities via metaproteomics. Nat. Commun..

[179] Machado V.S., Oikonomou G., Bicalho M.L., Knauer W.A., Gilbert R., Bicalho R.C. (2012). Investigation of postpartum dairy cows’ uterine microbial diversity using metagenomic pyrosequencing of the 16S rRNA gene. Vet. Microbiol..

[180] Winther A.R., Narvhus J.A., Smistad M., da Silva Duarte V., Bombelli A., Porcellato D. (2022). Longitudinal dynamics of the bovine udder microbiota. Anim. Microbiome.

[181] Myer P.R. (2019). Bovine Genome-Microbiome Interactions: Metagenomic Frontier for the Selection of Efficient Productivity in Cattle Systems. mSystems.

[182] Xue M.Y., Xie Y.Y., Zhong Y., Ma X.J., Sun H.Z., Liu J.X. (2022). Integrated meta-omics reveals new ruminal microbial features associated with feed efficiency in dairy cattle. Microbiome.

[183] Li F., Hitch T.C.A., Chen Y., Creevey C.J., Guan L.L. (2019). Comparative metagenomic and metatranscriptomic analyses reveal the breed effect on the rumen microbiome and its associations with feed efficiency in beef cattle. Microbiome.

[184] Hess M., Sczyrba A., Egan R., Kim T.W., Chokhawala H., Schroth G., Luo S., Clark D.S., Chen F., Zhang T. (2011). Metagenomic discovery of biomass-degrading genes and genomes from cow rumen. Science.

[185] Yu K., Zhang T. (2012). Metagenomic and metatranscriptomic analysis of microbial community structure and gene expression of activated sludge. PLoS ONE.

[186] Pitta D.W., Kumar S., Vecchiarelli B., Shirley D.J., Bittinger K., Baker L.D., Ferguson J.D., Thomsen N. (2014). Temporal dynamics in the ruminal microbiome of dairy cows during the transition period. J. Anim. Sci..

[187] Andersen T.O., Kunath B.J., Hagen L.H., Arntzen M.O., Pope P.B. (2021). Rumen metaproteomics: Closer to linking rumen microbial function to animal productivity traits. Methods.

[188] Van Den Bossche T., Arntzen M.O., Becher D., Benndorf D., Eijsink V.G.H., Henry C., Jagtap P.D., Jehmlich N., Juste C., Kunath B.J. (2021). The Metaproteomics Initiative: A coordinated approach for propelling the functional characterization of microbiomes. Microbiome.

[189] Maron P.A., Ranjard L., Mougel C., Lemanceau P. (2007). Metaproteomics: A new approach for studying functional microbial ecology. Microb. Ecol..

[190] Salvato F., Hettich R.L., Kleiner M. (2021). Five key aspects of metaproteomics as a tool to understand functional interactions in host-associated microbiomes. PLoS Pathog..

[191] Saleem F., Ametaj B.N., Bouatra S., Mandal R., Zebeli Q., Dunn S.M., Wishart D.S. (2012). A metabolomics approach to uncover the effects of grain diets on rumen health in dairy cows. J. Dairy Sci..

[192] Marrella M.A., Biase F.H. (2023). A multi-omics analysis identifies molecular features associated with fertility in heifers (*Bos taurus*). Sci. Rep..

[193] Kamada N., Chen G.Y., Inohara N., Nunez G. (2013). Control of pathogens and pathobionts by the gut microbiota. Nat. Immunol..

[194] Ma Z., Lee S., Jeong K.C. (2019). Mitigating antibiotic resistance at the livestock-environment interface: A review. J. Microbiol. Biotechnol..

[195] Hommerich K., Ruddat I., Hartmann M., Werner N., Kasbohrer A., Kreienbrock L. (2019). Monitoring Antibiotic Usage in German Dairy and Beef Cattle Farms-A Longitudinal Analysis. Front. Vet. Sci..

[196] Sommer F., Backhed F. (2013). The gut microbiota–masters of host development and physiology. Nat. Rev. Microbiol..

[197] Mowat A.M., Agace W.W. (2014). Regional specialization within the intestinal immune system. Nat. Rev. Immunol..

[198] Zangirolamo A.F., Souza A.K., Yokomizo D.N., Miguel A.K.A., Costa M.C.D., Alfieri A.A., Seneda M.M. (2024). Updates and Current Challenges in Reproductive Microbiome: A Comparative Analysis between Cows and Women. Animals.

[199] Rani K., Kaur G., Ali S.A. (2023). Probiotic-prebiotic therapeutic potential: A new horizon of microbial biotherapy to reduce female reproductive complications. PharmaNutrition.

[200] Reinoso-Pelaez E.L., Saura M., Gonzalez-Recio O., Gonzalez C., Fernandez A., Peiro-Pastor R., Lopez-Garcia A., Saborio-Montero A., Calvo J.H., Ramon M. (2023). Impact of oestrus synchronization devices on ewes vaginal microbiota and artificial insemination outcome. Front. Microbiol..

